# Measurements of top-quark pair differential cross-sections in the $e\mu$ channel in *pp* collisions at $\sqrt{s} = 13$ TeV using the ATLAS detector

**DOI:** 10.1140/epjc/s10052-017-4821-x

**Published:** 2017-05-08

**Authors:** M. Aaboud, G. Aad, B. Abbott, J. Abdallah, O. Abdinov, B. Abeloos, R. Aben, O. S. AbouZeid, N. L. Abraham, H. Abramowicz, H. Abreu, R. Abreu, Y. Abulaiti, B. S. Acharya, S. Adachi, L. Adamczyk, D. L. Adams, J. Adelman, S. Adomeit, T. Adye, A. A. Affolder, T. Agatonovic-Jovin, J. A. Aguilar-Saavedra, S. P. Ahlen, F. Ahmadov, G. Aielli, H. Akerstedt, T. P. A. Åkesson, A. V. Akimov, G. L. Alberghi, J. Albert, S. Albrand, M. J. Alconada Verzini, M. Aleksa, I. N. Aleksandrov, C. Alexa, G. Alexander, T. Alexopoulos, M. Alhroob, B. Ali, M. Aliev, G. Alimonti, J. Alison, S. P. Alkire, B. M. M. Allbrooke, B. W. Allen, P. P. Allport, A. Aloisio, A. Alonso, F. Alonso, C. Alpigiani, A. A. Alshehri, M. Alstaty, B. Alvarez Gonzalez, D. Álvarez Piqueras, M. G. Alviggi, B. T. Amadio, K. Amako, Y. Amaral Coutinho, C. Amelung, D. Amidei, S. P. Amor Dos Santos, A. Amorim, S. Amoroso, G. Amundsen, C. Anastopoulos, L. S. Ancu, N. Andari, T. Andeen, C. F. Anders, G. Anders, J. K. Anders, K. J. Anderson, A. Andreazza, V. Andrei, S. Angelidakis, I. Angelozzi, A. Angerami, F. Anghinolfi, A. V. Anisenkov, N. Anjos, A. Annovi, C. Antel, M. Antonelli, A. Antonov, F. Anulli, M. Aoki, L. Aperio Bella, G. Arabidze, Y. Arai, J. P. Araque, A. T. H. Arce, F. A. Arduh, J-F. Arguin, S. Argyropoulos, M. Arik, A. J. Armbruster, L. J. Armitage, O. Arnaez, H. Arnold, M. Arratia, O. Arslan, A. Artamonov, G. Artoni, S. Artz, S. Asai, N. Asbah, A. Ashkenazi, B. Åsman, L. Asquith, K. Assamagan, R. Astalos, M. Atkinson, N. B. Atlay, K. Augsten, G. Avolio, B. Axen, M. K. Ayoub, G. Azuelos, M. A. Baak, A. E. Baas, M. J. Baca, H. Bachacou, K. Bachas, M. Backes, M. Backhaus, P. Bagiacchi, P. Bagnaia, Y. Bai, J. T. Baines, O. K. Baker, E. M. Baldin, P. Balek, T. Balestri, F. Balli, W. K. Balunas, E. Banas, Sw. Banerjee, A. A. E. Bannoura, L. Barak, E. L. Barberio, D. Barberis, M. Barbero, T. Barillari, M-S. Barisits, T. Barklow, N. Barlow, S. L. Barnes, B. M. Barnett, R. M. Barnett, Z. Barnovska-Blenessy, A. Baroncelli, G. Barone, A. J. Barr, L. Barranco Navarro, F. Barreiro, J. Barreiro Guimarães da Costa, R. Bartoldus, A. E. Barton, P. Bartos, A. Basalaev, A. Bassalat, R. L. Bates, S. J. Batista, J. R. Batley, M. Battaglia, M. Bauce, F. Bauer, H. S. Bawa, J. B. Beacham, M. D. Beattie, T. Beau, P. H. Beauchemin, P. Bechtle, H. P. Beck, K. Becker, M. Becker, M. Beckingham, C. Becot, A. J. Beddall, A. Beddall, V. A. Bednyakov, M. Bedognetti, C. P. Bee, L. J. Beemster, T. A. Beermann, M. Begel, J. K. Behr, C. Belanger-Champagne, A. S. Bell, G. Bella, L. Bellagamba, A. Bellerive, M. Bellomo, K. Belotskiy, O. Beltramello, N. L. Belyaev, O. Benary, D. Benchekroun, M. Bender, K. Bendtz, N. Benekos, Y. Benhammou, E. Benhar Noccioli, J. Benitez, D. P. Benjamin, J. R. Bensinger, S. Bentvelsen, L. Beresford, M. Beretta, D. Berge, E. Bergeaas Kuutmann, N. Berger, J. Beringer, S. Berlendis, N. R. Bernard, C. Bernius, F. U. Bernlochner, T. Berry, P. Berta, C. Bertella, G. Bertoli, F. Bertolucci, I. A. Bertram, C. Bertsche, D. Bertsche, G. J. Besjes, O. Bessidskaia Bylund, M. Bessner, N. Besson, C. Betancourt, A. Bethani, S. Bethke, A. J. Bevan, R. M. Bianchi, L. Bianchini, M. Bianco, O. Biebel, D. Biedermann, R. Bielski, N. V. Biesuz, M. Biglietti, J. Bilbao De Mendizabal, T. R. V. Billoud, H. Bilokon, M. Bindi, S. Binet, A. Bingul, C. Bini, S. Biondi, T. Bisanz, D. M. Bjergaard, C. W. Black, J. E. Black, K. M. Black, D. Blackburn, R. E. Blair, J.-B. Blanchard, T. Blazek, I. Bloch, C. Blocker, A. Blue, W. Blum, U. Blumenschein, S. Blunier, G. J. Bobbink, V. S. Bobrovnikov, S. S. Bocchetta, A. Bocci, C. Bock, M. Boehler, D. Boerner, J. A. Bogaerts, D. Bogavac, A. G. Bogdanchikov, C. Bohm, V. Boisvert, P. Bokan, T. Bold, A. S. Boldyrev, M. Bomben, M. Bona, M. Boonekamp, A. Borisov, G. Borissov, J. Bortfeldt, D. Bortoletto, V. Bortolotto, K. Bos, D. Boscherini, M. Bosman, J. D. Bossio Sola, J. Boudreau, J. Bouffard, E. V. Bouhova-Thacker, D. Boumediene, C. Bourdarios, S. K. Boutle, A. Boveia, J. Boyd, I. R. Boyko, J. Bracinik, A. Brandt, G. Brandt, O. Brandt, U. Bratzler, B. Brau, J. E. Brau, W. D. Breaden Madden, K. Brendlinger, A. J. Brennan, L. Brenner, R. Brenner, S. Bressler, T. M. Bristow, D. Britton, D. Britzger, F. M. Brochu, I. Brock, R. Brock, G. Brooijmans, T. Brooks, W. K. Brooks, J. Brosamer, E. Brost, J. H Broughton, P. A. Bruckman de Renstrom, D. Bruncko, R. Bruneliere, A. Bruni, G. Bruni, L. S. Bruni, BH. Brunt, M. Bruschi, N. Bruscino, P. Bryant, L. Bryngemark, T. Buanes, Q. Buat, P. Buchholz, A. G. Buckley, I. A. Budagov, F. Buehrer, M. K. Bugge, O. Bulekov, D. Bullock, H. Burckhart, S. Burdin, C. D. Burgard, B. Burghgrave, K. Burka, S. Burke, I. Burmeister, J. T. P. Burr, E. Busato, D. Büscher, V. Büscher, P. Bussey, J. M. Butler, C. M. Buttar, J. M. Butterworth, P. Butti, W. Buttinger, A. Buzatu, A. R. Buzykaev, S. Cabrera Urbán, D. Caforio, V. M. Cairo, O. Cakir, N. Calace, P. Calafiura, A. Calandri, G. Calderini, P. Calfayan, G. Callea, L. P. Caloba, S. Calvente Lopez, D. Calvet, S. Calvet, T. P. Calvet, R. Camacho Toro, S. Camarda, P. Camarri, D. Cameron, R. Caminal Armadans, C. Camincher, S. Campana, M. Campanelli, A. Camplani, A. Campoverde, V. Canale, A. Canepa, M. Cano Bret, J. Cantero, T. Cao, M. D. M. Capeans Garrido, I. Caprini, M. Caprini, M. Capua, R. M. Carbone, R. Cardarelli, F. Cardillo, I. Carli, T. Carli, G. Carlino, L. Carminati, R. M. D. Carney, S. Caron, E. Carquin, G. D. Carrillo-Montoya, J. R. Carter, J. Carvalho, D. Casadei, M. P. Casado, M. Casolino, D. W. Casper, E. Castaneda-Miranda, R. Castelijn, A. Castelli, V. Castillo Gimenez, N. F. Castro, A. Catinaccio, J. R. Catmore, A. Cattai, J. Caudron, V. Cavaliere, E. Cavallaro, D. Cavalli, M. Cavalli-Sforza, V. Cavasinni, F. Ceradini, L. Cerda Alberich, A. S. Cerqueira, A. Cerri, L. Cerrito, F. Cerutti, M. Cerv, A. Cervelli, S. A. Cetin, A. Chafaq, D. Chakraborty, S. K. Chan, Y. L. Chan, P. Chang, J. D. Chapman, D. G. Charlton, A. Chatterjee, C. C. Chau, C. A. Chavez Barajas, S. Che, S. Cheatham, A. Chegwidden, S. Chekanov, S. V. Chekulaev, G. A. Chelkov, M. A. Chelstowska, C. Chen, H. Chen, K. Chen, S. Chen, S. Chen, X. Chen, Y. Chen, H. C. Cheng, H. J Cheng, Y. Cheng, A. Cheplakov, E. Cheremushkina, R. Cherkaoui El Moursli, V. Chernyatin, E. Cheu, L. Chevalier, V. Chiarella, G. Chiarelli, G. Chiodini, A. S. Chisholm, A. Chitan, M. V. Chizhov, K. Choi, A. R. Chomont, S. Chouridou, B. K. B. Chow, V. Christodoulou, D. Chromek-Burckhart, J. Chudoba, A. J. Chuinard, J. J. Chwastowski, L. Chytka, G. Ciapetti, A. K. Ciftci, D. Cinca, V. Cindro, I. A. Cioara, C. Ciocca, A. Ciocio, F. Cirotto, Z. H. Citron, M. Citterio, M. Ciubancan, A. Clark, B. L. Clark, M. R. Clark, P. J. Clark, R. N. Clarke, C. Clement, Y. Coadou, M. Cobal, A. Coccaro, J. Cochran, L. Colasurdo, B. Cole, A. P. Colijn, J. Collot, T. Colombo, G. Compostella, P. Conde Muiño, E. Coniavitis, S. H. Connell, I. A. Connelly, V. Consorti, S. Constantinescu, G. Conti, F. Conventi, M. Cooke, B. D. Cooper, A. M. Cooper-Sarkar, K. J. R. Cormier, T. Cornelissen, M. Corradi, F. Corriveau, A. Cortes-Gonzalez, G. Cortiana, G. Costa, M. J. Costa, D. Costanzo, G. Cottin, G. Cowan, B. E. Cox, K. Cranmer, S. J. Crawley, G. Cree, S. Crépé-Renaudin, F. Crescioli, W. A. Cribbs, M. Crispin Ortuzar, M. Cristinziani, V. Croft, G. Crosetti, A. Cueto, T. Cuhadar Donszelmann, J. Cummings, M. Curatolo, J. Cúth, H. Czirr, P. Czodrowski, G. D’amen, S. D’Auria, M. D’Onofrio, M. J. Da Cunha Sargedas De Sousa, C. Da Via, W. Dabrowski, T. Dado, T. Dai, O. Dale, F. Dallaire, C. Dallapiccola, M. Dam, J. R. Dandoy, N. P. Dang, A. C. Daniells, N. S. Dann, M. Danninger, M. Dano Hoffmann, V. Dao, G. Darbo, S. Darmora, J. Dassoulas, A. Dattagupta, W. Davey, C. David, T. Davidek, M. Davies, P. Davison, E. Dawe, I. Dawson, K. De, R. de Asmundis, A. De Benedetti, S. De Castro, S. De Cecco, N. De Groot, P. de Jong, H. De la Torre, F. De Lorenzi, A. De Maria, D. De Pedis, A. De Salvo, U. De Sanctis, A. De Santo, J. B. De Vivie De Regie, W. J. Dearnaley, R. Debbe, C. Debenedetti, D. V. Dedovich, N. Dehghanian, I. Deigaard, M. Del Gaudio, J. Del Peso, T. Del Prete, D. Delgove, F. Deliot, C. M. Delitzsch, A. Dell’Acqua, L. Dell’Asta, M. Dell’Orso, M. Della Pietra, D. della Volpe, M. Delmastro, P. A. Delsart, D. A. DeMarco, S. Demers, M. Demichev, A. Demilly, S. P. Denisov, D. Denysiuk, D. Derendarz, J. E. Derkaoui, F. Derue, P. Dervan, K. Desch, C. Deterre, K. Dette, P. O. Deviveiros, A. Dewhurst, S. Dhaliwal, A. Di Ciaccio, L. Di Ciaccio, W. K. Di Clemente, C. Di Donato, A. Di Girolamo, B. Di Girolamo, B. Di Micco, R. Di Nardo, A. Di Simone, R. Di Sipio, D. Di Valentino, C. Diaconu, M. Diamond, F. A. Dias, M. A. Diaz, E. B. Diehl, J. Dietrich, S. Díez Cornell, A. Dimitrievska, J. Dingfelder, P. Dita, S. Dita, F. Dittus, F. Djama, T. Djobava, J. I. Djuvsland, M. A. B. do Vale, D. Dobos, M. Dobre, C. Doglioni, J. Dolejsi, Z. Dolezal, M. Donadelli, S. Donati, P. Dondero, J. Donini, J. Dopke, A. Doria, M. T. Dova, A. T. Doyle, E. Drechsler, M. Dris, Y. Du, J. Duarte-Campderros, E. Duchovni, G. Duckeck, O. A. Ducu, D. Duda, A. Dudarev, A. Chr. Dudder, E. M. Duffield, L. Duflot, M. Dührssen, M. Dumancic, M. Dunford, H. Duran Yildiz, M. Düren, A. Durglishvili, D. Duschinger, B. Dutta, M. Dyndal, C. Eckardt, K. M. Ecker, R. C. Edgar, N. C. Edwards, T. Eifert, G. Eigen, K. Einsweiler, T. Ekelof, M. El Kacimi, V. Ellajosyula, M. Ellert, S. Elles, F. Ellinghaus, A. A. Elliot, N. Ellis, J. Elmsheuser, M. Elsing, D. Emeliyanov, Y. Enari, O. C. Endner, J. S. Ennis, J. Erdmann, A. Ereditato, G. Ernis, J. Ernst, M. Ernst, S. Errede, E. Ertel, M. Escalier, H. Esch, C. Escobar, B. Esposito, A. I. Etienvre, E. Etzion, H. Evans, A. Ezhilov, M. Ezzi, F. Fabbri, L. Fabbri, G. Facini, R. M. Fakhrutdinov, S. Falciano, R. J. Falla, J. Faltova, Y. Fang, M. Fanti, A. Farbin, A. Farilla, C. Farina, E. M. Farina, T. Farooque, S. Farrell, S. M. Farrington, P. Farthouat, F. Fassi, P. Fassnacht, D. Fassouliotis, M. Faucci Giannelli, A. Favareto, W. J. Fawcett, L. Fayard, O. L. Fedin, W. Fedorko, S. Feigl, L. Feligioni, C. Feng, E. J. Feng, H. Feng, A. B. Fenyuk, L. Feremenga, P. Fernandez Martinez, S. Fernandez Perez, J. Ferrando, A. Ferrari, P. Ferrari, R. Ferrari, D. E. Ferreira de Lima, A. Ferrer, D. Ferrere, C. Ferretti, A. Ferretto Parodi, F. Fiedler, A. Filipčič, M. Filipuzzi, F. Filthaut, M. Fincke-Keeler, K. D. Finelli, M. C. N. Fiolhais, L. Fiorini, A. Firan, A. Fischer, C. Fischer, J. Fischer, W. C. Fisher, N. Flaschel, I. Fleck, P. Fleischmann, G. T. Fletcher, R. R. M. Fletcher, T. Flick, L. R. Flores Castillo, M. J. Flowerdew, G. T. Forcolin, A. Formica, A. Forti, A. G. Foster, D. Fournier, H. Fox, S. Fracchia, P. Francavilla, M. Franchini, D. Francis, L. Franconi, M. Franklin, M. Frate, M. Fraternali, D. Freeborn, S. M. Fressard-Batraneanu, F. Friedrich, D. Froidevaux, J. A. Frost, C. Fukunaga, E. Fullana Torregrosa, T. Fusayasu, J. Fuster, C. Gabaldon, O. Gabizon, A. Gabrielli, A. Gabrielli, G. P. Gach, S. Gadatsch, S. Gadomski, G. Gagliardi, L. G. Gagnon, P. Gagnon, C. Galea, B. Galhardo, E. J. Gallas, B. J. Gallop, P. Gallus, G. Galster, K. K. Gan, S. Ganguly, J. Gao, Y. Gao, Y. S. Gao, F. M. Garay Walls, C. García, J. E. García Navarro, M. Garcia-Sciveres, R. W. Gardner, N. Garelli, V. Garonne, A. Gascon Bravo, K. Gasnikova, C. Gatti, A. Gaudiello, G. Gaudio, L. Gauthier, I. L. Gavrilenko, C. Gay, G. Gaycken, E. N. Gazis, Z. Gecse, C. N. P. Gee, Ch. Geich-Gimbel, M. Geisen, M. P. Geisler, K. Gellerstedt, C. Gemme, M. H. Genest, C. Geng, S. Gentile, C. Gentsos, S. George, D. Gerbaudo, A. Gershon, S. Ghasemi, M. Ghneimat, B. Giacobbe, S. Giagu, P. Giannetti, B. Gibbard, S. M. Gibson, M. Gignac, M. Gilchriese, T. P. S. Gillam, D. Gillberg, G. Gilles, D. M. Gingrich, N. Giokaris, M. P. Giordani, F. M. Giorgi, F. M. Giorgi, P. F. Giraud, P. Giromini, D. Giugni, F. Giuli, C. Giuliani, M. Giulini, B. K. Gjelsten, S. Gkaitatzis, I. Gkialas, E. L. Gkougkousis, L. K. Gladilin, C. Glasman, J. Glatzer, P. C. F. Glaysher, A. Glazov, M. Goblirsch-Kolb, J. Godlewski, S. Goldfarb, T. Golling, D. Golubkov, A. Gomes, R. Gonçalo, J. Goncalves Pinto Firmino Da Costa, G. Gonella, L. Gonella, A. Gongadze, S. González de la Hoz, S. Gonzalez-Sevilla, L. Goossens, P. A. Gorbounov, H. A. Gordon, I. Gorelov, B. Gorini, E. Gorini, A. Gorišek, E. Gornicki, A. T. Goshaw, C. Gössling, M. I. Gostkin, C. R. Goudet, D. Goujdami, A. G. Goussiou, N. Govender, E. Gozani, L. Graber, I. Grabowska-Bold, P. O. J. Gradin, P. Grafström, J. Gramling, E. Gramstad, S. Grancagnolo, V. Gratchev, P. M. Gravila, H. M. Gray, E. Graziani, Z. D. Greenwood, C. Grefe, K. Gregersen, I. M. Gregor, P. Grenier, K. Grevtsov, J. Griffiths, A. A. Grillo, K. Grimm, S. Grinstein, Ph. Gris, J.-F. Grivaz, S. Groh, E. Gross, J. Grosse-Knetter, G. C. Grossi, Z. J. Grout, L. Guan, W. Guan, J. Guenther, F. Guescini, D. Guest, O. Gueta, B. Gui, E. Guido, T. Guillemin, S. Guindon, U. Gul, C. Gumpert, J. Guo, Y. Guo, R. Gupta, S. Gupta, G. Gustavino, P. Gutierrez, N. G. Gutierrez Ortiz, C. Gutschow, C. Guyot, C. Gwenlan, C. B. Gwilliam, A. Haas, C. Haber, H. K. Hadavand, N. Haddad, A. Hadef, S. Hageböck, M. Hagihara, Z. Hajduk, H. Hakobyan, M. Haleem, J. Haley, G. Halladjian, G. D. Hallewell, K. Hamacher, P. Hamal, K. Hamano, A. Hamilton, G. N. Hamity, P. G. Hamnett, L. Han, K. Hanagaki, K. Hanawa, M. Hance, B. Haney, P. Hanke, R. Hanna, J. B. Hansen, J. D. Hansen, M. C. Hansen, P. H. Hansen, K. Hara, A. S. Hard, T. Harenberg, F. Hariri, S. Harkusha, R. D. Harrington, P. F. Harrison, F. Hartjes, N. M. Hartmann, M. Hasegawa, Y. Hasegawa, A. Hasib, S. Hassani, S. Haug, R. Hauser, L. Hauswald, M. Havranek, C. M. Hawkes, R. J. Hawkings, D. Hayakawa, D. Hayden, C. P. Hays, J. M. Hays, H. S. Hayward, S. J. Haywood, S. J. Head, T. Heck, V. Hedberg, L. Heelan, S. Heim, T. Heim, B. Heinemann, J. J. Heinrich, L. Heinrich, C. Heinz, J. Hejbal, L. Helary, S. Hellman, C. Helsens, J. Henderson, R. C. W. Henderson, Y. Heng, S. Henkelmann, A. M. Henriques Correia, S. Henrot-Versille, G. H. Herbert, H. Herde, V. Herget, Y. Hernández Jiménez, G. Herten, R. Hertenberger, L. Hervas, G. G. Hesketh, N. P. Hessey, J. W. Hetherly, R. Hickling, E. Higón-Rodriguez, E. Hill, J. C. Hill, K. H. Hiller, S. J. Hillier, I. Hinchliffe, E. Hines, R. R. Hinman, M. Hirose, D. Hirschbuehl, J. Hobbs, N. Hod, M. C. Hodgkinson, P. Hodgson, A. Hoecker, M. R. Hoeferkamp, F. Hoenig, D. Hohn, T. R. Holmes, M. Homann, T. Honda, T. M. Hong, B. H. Hooberman, W. H. Hopkins, Y. Horii, A. J. Horton, J-Y. Hostachy, S. Hou, A. Hoummada, J. Howarth, J. Hoya, M. Hrabovsky, I. Hristova, J. Hrivnac, T. Hryn’ova, A. Hrynevich, C. Hsu, P. J. Hsu, S.-C. Hsu, Q. Hu, S. Hu, Y. Huang, Z. Hubacek, F. Hubaut, F. Huegging, T. B. Huffman, E. W. Hughes, G. Hughes, M. Huhtinen, P. Huo, N. Huseynov, J. Huston, J. Huth, G. Iacobucci, G. Iakovidis, I. Ibragimov, L. Iconomidou-Fayard, E. Ideal, Z. Idrissi, P. Iengo, O. Igonkina, T. Iizawa, Y. Ikegami, M. Ikeno, Y. Ilchenko, D. Iliadis, N. Ilic, T. Ince, G. Introzzi, P. Ioannou, M. Iodice, K. Iordanidou, V. Ippolito, N. Ishijima, M. Ishino, M. Ishitsuka, R. Ishmukhametov, C. Issever, S. Istin, F. Ito, J. M. Iturbe Ponce, R. Iuppa, W. Iwanski, H. Iwasaki, J. M. Izen, V. Izzo, S. Jabbar, B. Jackson, P. Jackson, V. Jain, K. B. Jakobi, K. Jakobs, S. Jakobsen, T. Jakoubek, D. O. Jamin, D. K. Jana, R. Jansky, J. Janssen, M. Janus, G. Jarlskog, N. Javadov, T. Javůrek, F. Jeanneau, L. Jeanty, G.-Y. Jeng, D. Jennens, P. Jenni, C. Jeske, S. Jézéquel, H. Ji, J. Jia, H. Jiang, Y. Jiang, Z. Jiang, S. Jiggins, J. Jimenez Pena, S. Jin, A. Jinaru, O. Jinnouchi, H. Jivan, P. Johansson, K. A. Johns, W. J. Johnson, K. Jon-And, G. Jones, R. W. L. Jones, S. Jones, T. J. Jones, J. Jongmanns, P. M. Jorge, J. Jovicevic, X. Ju, A. Juste Rozas, M. K. Köhler, A. Kaczmarska, M. Kado, H. Kagan, M. Kagan, S. J. Kahn, T. Kaji, E. Kajomovitz, C. W. Kalderon, A. Kaluza, S. Kama, A. Kamenshchikov, N. Kanaya, S. Kaneti, L. Kanjir, V. A. Kantserov, J. Kanzaki, B. Kaplan, L. S. Kaplan, A. Kapliy, D. Kar, K. Karakostas, A. Karamaoun, N. Karastathis, M. J. Kareem, E. Karentzos, M. Karnevskiy, S. N. Karpov, Z. M. Karpova, K. Karthik, V. Kartvelishvili, A. N. Karyukhin, K. Kasahara, L. Kashif, R. D. Kass, A. Kastanas, Y. Kataoka, C. Kato, A. Katre, J. Katzy, K. Kawade, K. Kawagoe, T. Kawamoto, G. Kawamura, V. F. Kazanin, R. Keeler, R. Kehoe, J. S. Keller, J. J. Kempster, H. Keoshkerian, O. Kepka, B. P. Kerševan, S. Kersten, R. A. Keyes, M. Khader, F. Khalil-zada, A. Khanov, A. G. Kharlamov, T. Kharlamova, T. J. Khoo, V. Khovanskiy, E. Khramov, J. Khubua, S. Kido, C. R. Kilby, H. Y. Kim, S. H. Kim, Y. K. Kim, N. Kimura, O. M. Kind, B. T. King, M. King, J. Kirk, A. E. Kiryunin, T. Kishimoto, D. Kisielewska, F. Kiss, K. Kiuchi, O. Kivernyk, E. Kladiva, M. H. Klein, M. Klein, U. Klein, K. Kleinknecht, P. Klimek, A. Klimentov, R. Klingenberg, J. A. Klinger, T. Klioutchnikova, E.-E. Kluge, P. Kluit, S. Kluth, J. Knapik, E. Kneringer, E. B. F. G. Knoops, A. Knue, A. Kobayashi, D. Kobayashi, T. Kobayashi, M. Kobel, M. Kocian, P. Kodys, T. Koffas, E. Koffeman, N. M. Köhler, T. Koi, H. Kolanoski, M. Kolb, I. Koletsou, A. A. Komar, Y. Komori, T. Kondo, N. Kondrashova, K. Köneke, A. C. König, T. Kono, R. Konoplich, N. Konstantinidis, R. Kopeliansky, S. Koperny, L. Köpke, A. K. Kopp, K. Korcyl, K. Kordas, A. Korn, A. A. Korol, I. Korolkov, E. V. Korolkova, O. Kortner, S. Kortner, T. Kosek, V. V. Kostyukhin, A. Kotwal, A. Koulouris, A. Kourkoumeli-Charalampidi, C. Kourkoumelis, V. Kouskoura, A. B. Kowalewska, R. Kowalewski, T. Z. Kowalski, C. Kozakai, W. Kozanecki, A. S. Kozhin, V. A. Kramarenko, G. Kramberger, D. Krasnopevtsev, M. W. Krasny, A. Krasznahorkay, A. Kravchenko, M. Kretz, J. Kretzschmar, K. Kreutzfeldt, P. Krieger, K. Krizka, K. Kroeninger, H. Kroha, J. Kroll, J. Kroseberg, J. Krstic, U. Kruchonak, H. Krüger, N. Krumnack, M. C. Kruse, M. Kruskal, T. Kubota, H. Kucuk, S. Kuday, J. T. Kuechler, S. Kuehn, A. Kugel, F. Kuger, A. Kuhl, T. Kuhl, V. Kukhtin, R. Kukla, Y. Kulchitsky, S. Kuleshov, M. Kuna, T. Kunigo, A. Kupco, H. Kurashige, Y. A. Kurochkin, V. Kus, E. S. Kuwertz, M. Kuze, J. Kvita, T. Kwan, D. Kyriazopoulos, A. La Rosa, J. L. La Rosa Navarro, L. La Rotonda, C. Lacasta, F. Lacava, J. Lacey, H. Lacker, D. Lacour, V. R. Lacuesta, E. Ladygin, R. Lafaye, B. Laforge, T. Lagouri, S. Lai, S. Lammers, W. Lampl, E. Lançon, U. Landgraf, M. P. J. Landon, M. C. Lanfermann, V. S. Lang, J. C. Lange, A. J. Lankford, F. Lanni, K. Lantzsch, A. Lanza, S. Laplace, C. Lapoire, J. F. Laporte, T. Lari, F. Lasagni Manghi, M. Lassnig, P. Laurelli, W. Lavrijsen, A. T. Law, P. Laycock, T. Lazovich, M. Lazzaroni, B. Le, O. Le Dortz, E. Le Guirriec, E. P. Le Quilleuc, M. LeBlanc, T. LeCompte, F. Ledroit-Guillon, C. A. Lee, S. C. Lee, L. Lee, B. Lefebvre, G. Lefebvre, M. Lefebvre, F. Legger, C. Leggett, A. Lehan, G. Lehmann Miotto, X. Lei, W. A. Leight, A. G. Leister, M. A. L. Leite, R. Leitner, D. Lellouch, B. Lemmer, K. J. C. Leney, T. Lenz, B. Lenzi, R. Leone, S. Leone, C. Leonidopoulos, S. Leontsinis, G. Lerner, C. Leroy, A. A. J. Lesage, C. G. Lester, M. Levchenko, J. Levêque, D. Levin, L. J. Levinson, M. Levy, D. Lewis, A. M. Leyko, M. Leyton, B. Li, C. Li, H. Li, H. L. Li, L. Li, L. Li, Q. Li, S. Li, X. Li, Y. Li, Z. Liang, B. Liberti, A. Liblong, P. Lichard, K. Lie, J. Liebal, W. Liebig, A. Limosani, S. C. Lin, T. H. Lin, B. E. Lindquist, A. E. Lionti, E. Lipeles, A. Lipniacka, M. Lisovyi, T. M. Liss, A. Lister, A. M. Litke, B. Liu, D. Liu, H. Liu, H. Liu, J. Liu, J. B. Liu, K. Liu, L. Liu, M. Liu, M. Liu, Y. L. Liu, Y. Liu, M. Livan, A. Lleres, J. Llorente Merino, S. L. Lloyd, F. Lo Sterzo, E. M. Lobodzinska, P. Loch, F. K. Loebinger, K. M. Loew, A. Loginov, T. Lohse, K. Lohwasser, M. Lokajicek, B. A. Long, J. D. Long, R. E. Long, L. Longo, K. A. Looper, J. A. Lopez Lopez, D. Lopez Mateos, B. Lopez Paredes, I. Lopez Paz, A. Lopez Solis, J. Lorenz, N. Lorenzo Martinez, M. Losada, P. J. Lösel, X. Lou, A. Lounis, J. Love, P. A. Love, H. Lu, N. Lu, H. J. Lubatti, C. Luci, A. Lucotte, C. Luedtke, F. Luehring, W. Lukas, L. Luminari, O. Lundberg, B. Lund-Jensen, P. M. Luzi, D. Lynn, R. Lysak, E. Lytken, V. Lyubushkin, H. Ma, L. L. Ma, Y. Ma, G. Maccarrone, A. Macchiolo, C. M. Macdonald, B. Maček, J. Machado Miguens, D. Madaffari, R. Madar, H. J. Maddocks, W. F. Mader, A. Madsen, J. Maeda, S. Maeland, T. Maeno, A. Maevskiy, E. Magradze, J. Mahlstedt, C. Maiani, C. Maidantchik, A. A. Maier, T. Maier, A. Maio, S. Majewski, Y. Makida, N. Makovec, B. Malaescu, Pa. Malecki, V. P. Maleev, F. Malek, U. Mallik, D. Malon, C. Malone, C. Malone, S. Maltezos, S. Malyukov, J. Mamuzic, G. Mancini, L. Mandelli, I. Mandić, J. Maneira, L. Manhaes de Andrade Filho, J. Manjarres Ramos, A. Mann, A. Manousos, B. Mansoulie, J. D. Mansour, R. Mantifel, M. Mantoani, S. Manzoni, L. Mapelli, G. Marceca, L. March, G. Marchiori, M. Marcisovsky, M. Marjanovic, D. E. Marley, F. Marroquim, S. P. Marsden, Z. Marshall, S. Marti-Garcia, B. Martin, T. A. Martin, V. J. Martin, B. Martin dit Latour, M. Martinez, V. I. Martinez Outschoorn, S. Martin-Haugh, V. S. Martoiu, A. C. Martyniuk, A. Marzin, L. Masetti, T. Mashimo, R. Mashinistov, J. Masik, A. L. Maslennikov, I. Massa, L. Massa, P. Mastrandrea, A. Mastroberardino, T. Masubuchi, P. Mättig, J. Mattmann, J. Maurer, S. J. Maxfield, D. A. Maximov, R. Mazini, I. Maznas, S. M. Mazza, N. C. Mc Fadden, G. Mc Goldrick, S. P. Mc Kee, A. McCarn, R. L. McCarthy, T. G. McCarthy, L. I. McClymont, E. F. McDonald, J. A. Mcfayden, G. Mchedlidze, S. J. McMahon, R. A. McPherson, M. Medinnis, S. Meehan, S. Mehlhase, A. Mehta, K. Meier, C. Meineck, B. Meirose, D. Melini, B. R. Mellado Garcia, M. Melo, F. Meloni, X. T. Meng, A. Mengarelli, S. Menke, E. Meoni, S. Mergelmeyer, P. Mermod, L. Merola, C. Meroni, F. S. Merritt, A. Messina, J. Metcalfe, A. S. Mete, C. Meyer, C. Meyer, J-P. Meyer, J. Meyer, H. Meyer Zu Theenhausen, F. Miano, R. P. Middleton, S. Miglioranzi, L. Mijović, G. Mikenberg, M. Mikestikova, M. Mikuž, M. Milesi, A. Milic, D. W. Miller, C. Mills, A. Milov, D. A. Milstead, A. A. Minaenko, Y. Minami, I. A. Minashvili, A. I. Mincer, B. Mindur, M. Mineev, Y. Minegishi, Y. Ming, L. M. Mir, K. P. Mistry, T. Mitani, J. Mitrevski, V. A. Mitsou, A. Miucci, P. S. Miyagawa, J. U. Mjörnmark, M. Mlynarikova, T. Moa, K. Mochizuki, S. Mohapatra, S. Molander, R. Moles-Valls, R. Monden, M. C. Mondragon, K. Mönig, J. Monk, E. Monnier, A. Montalbano, J. Montejo Berlingen, F. Monticelli, S. Monzani, R. W. Moore, N. Morange, D. Moreno, M. Moreno Llácer, P. Morettini, S. Morgenstern, D. Mori, T. Mori, M. Morii, M. Morinaga, V. Morisbak, S. Moritz, A. K. Morley, G. Mornacchi, J. D. Morris, S. S. Mortensen, L. Morvaj, M. Mosidze, J. Moss, K. Motohashi, R. Mount, E. Mountricha, E. J. W. Moyse, S. Muanza, R. D. Mudd, F. Mueller, J. Mueller, R. S. P. Mueller, T. Mueller, D. Muenstermann, P. Mullen, G. A. Mullier, F. J. Munoz Sanchez, J. A. Murillo Quijada, W. J. Murray, H. Musheghyan, M. Muškinja, A. G. Myagkov, M. Myska, B. P. Nachman, O. Nackenhorst, K. Nagai, R. Nagai, K. Nagano, Y. Nagasaka, K. Nagata, M. Nagel, E. Nagy, A. M. Nairz, Y. Nakahama, K. Nakamura, T. Nakamura, I. Nakano, R. F. Naranjo Garcia, R. Narayan, D. I. Narrias Villar, I. Naryshkin, T. Naumann, G. Navarro, R. Nayyar, H. A. Neal, P. Yu. Nechaeva, T. J. Neep, A. Negri, M. Negrini, S. Nektarijevic, C. Nellist, A. Nelson, S. Nemecek, P. Nemethy, A. A. Nepomuceno, M. Nessi, M. S. Neubauer, M. Neumann, R. M. Neves, P. Nevski, P. R. Newman, D. H. Nguyen, T. Nguyen Manh, R. B. Nickerson, R. Nicolaidou, J. Nielsen, A. Nikiforov, V. Nikolaenko, I. Nikolic-Audit, K. Nikolopoulos, J. K. Nilsen, P. Nilsson, Y. Ninomiya, A. Nisati, R. Nisius, T. Nobe, M. Nomachi, I. Nomidis, T. Nooney, S. Norberg, M. Nordberg, N. Norjoharuddeen, O. Novgorodova, S. Nowak, M. Nozaki, L. Nozka, K. Ntekas, E. Nurse, F. Nuti, F. O’grady, D. C. O’Neil, A. A. O’Rourke, V. O’Shea, F. G. Oakham, H. Oberlack, T. Obermann, J. Ocariz, A. Ochi, I. Ochoa, J. P. Ochoa-Ricoux, S. Oda, S. Odaka, H. Ogren, A. Oh, S. H. Oh, C. C. Ohm, H. Ohman, H. Oide, H. Okawa, Y. Okumura, T. Okuyama, A. Olariu, L. F. Oleiro Seabra, S. A. Olivares Pino, D. Oliveira Damazio, A. Olszewski, J. Olszowska, A. Onofre, K. Onogi, P. U. E. Onyisi, M. J. Oreglia, Y. Oren, D. Orestano, N. Orlando, R. S. Orr, B. Osculati, R. Ospanov, G. Otero y Garzon, H. Otono, M. Ouchrif, F. Ould-Saada, A. Ouraou, K. P. Oussoren, Q. Ouyang, M. Owen, R. E. Owen, V. E. Ozcan, N. Ozturk, K. Pachal, A. Pacheco Pages, L. Pacheco Rodriguez, C. Padilla Aranda, M. Pagáčová, S. Pagan Griso, M. Paganini, F. Paige, P. Pais, K. Pajchel, G. Palacino, S. Palazzo, S. Palestini, M. Palka, D. Pallin, E. St. Panagiotopoulou, C. E. Pandini, J. G. Panduro Vazquez, P. Pani, S. Panitkin, D. Pantea, L. Paolozzi, Th. D. Papadopoulou, K. Papageorgiou, A. Paramonov, D. Paredes Hernandez, A. J. Parker, M. A. Parker, K. A. Parker, F. Parodi, J. A. Parsons, U. Parzefall, V. R. Pascuzzi, E. Pasqualucci, S. Passaggio, Fr. Pastore, G. Pásztor, S. Pataraia, J. R. Pater, T. Pauly, J. Pearce, B. Pearson, L. E. Pedersen, M. Pedersen, S. Pedraza Lopez, R. Pedro, S. V. Peleganchuk, O. Penc, C. Peng, H. Peng, J. Penwell, B. S. Peralva, M. M. Perego, D. V. Perepelitsa, E. Perez Codina, L. Perini, H. Pernegger, S. Perrella, R. Peschke, V. D. Peshekhonov, K. Peters, R. F. Y. Peters, B. A. Petersen, T. C. Petersen, E. Petit, A. Petridis, C. Petridou, P. Petroff, E. Petrolo, M. Petrov, F. Petrucci, N. E. Pettersson, A. Peyaud, R. Pezoa, P. W. Phillips, G. Piacquadio, E. Pianori, A. Picazio, E. Piccaro, M. Piccinini, M. A. Pickering, R. Piegaia, J. E. Pilcher, A. D. Pilkington, A. W. J. Pin, M. Pinamonti, J. L. Pinfold, A. Pingel, S. Pires, H. Pirumov, M. Pitt, L. Plazak, M.-A. Pleier, V. Pleskot, E. Plotnikova, P. Plucinski, D. Pluth, R. Poettgen, L. Poggioli, D. Pohl, G. Polesello, A. Poley, A. Policicchio, R. Polifka, A. Polini, C. S. Pollard, V. Polychronakos, K. Pommès, L. Pontecorvo, B. G. Pope, G. A. Popeneciu, A. Poppleton, S. Pospisil, K. Potamianos, I. N. Potrap, C. J. Potter, C. T. Potter, G. Poulard, J. Poveda, V. Pozdnyakov, M. E. Pozo Astigarraga, P. Pralavorio, A. Pranko, S. Prell, D. Price, L. E. Price, M. Primavera, S. Prince, K. Prokofiev, F. Prokoshin, S. Protopopescu, J. Proudfoot, M. Przybycien, D. Puddu, M. Purohit, P. Puzo, J. Qian, G. Qin, Y. Qin, A. Quadt, W. B. Quayle, M. Queitsch-Maitland, D. Quilty, S. Raddum, V. Radeka, V. Radescu, S. K. Radhakrishnan, P. Radloff, P. Rados, F. Ragusa, G. Rahal, J. A. Raine, S. Rajagopalan, M. Rammensee, C. Rangel-Smith, M. G. Ratti, D. M. Rauch, F. Rauscher, S. Rave, T. Ravenscroft, I. Ravinovich, M. Raymond, A. L. Read, N. P. Readioff, M. Reale, D. M. Rebuzzi, A. Redelbach, G. Redlinger, R. Reece, R. G. Reed, K. Reeves, L. Rehnisch, J. Reichert, A. Reiss, C. Rembser, H. Ren, M. Rescigno, S. Resconi, O. L. Rezanova, P. Reznicek, R. Rezvani, R. Richter, S. Richter, E. Richter-Was, O. Ricken, M. Ridel, P. Rieck, C. J. Riegel, J. Rieger, O. Rifki, M. Rijssenbeek, A. Rimoldi, M. Rimoldi, L. Rinaldi, B. Ristić, E. Ritsch, I. Riu, F. Rizatdinova, E. Rizvi, C. Rizzi, S. H. Robertson, A. Robichaud-Veronneau, D. Robinson, J. E. M. Robinson, A. Robson, C. Roda, Y. Rodina, A. Rodriguez Perez, D. Rodriguez Rodriguez, S. Roe, C. S. Rogan, O. Røhne, J. Roloff, A. Romaniouk, M. Romano, S. M. Romano Saez, E. Romero Adam, N. Rompotis, M. Ronzani, L. Roos, E. Ros, S. Rosati, K. Rosbach, P. Rose, N.-A. Rosien, V. Rossetti, E. Rossi, L. P. Rossi, J. H. N. Rosten, R. Rosten, M. Rotaru, I. Roth, J. Rothberg, D. Rousseau, A. Rozanov, Y. Rozen, X. Ruan, F. Rubbo, M. S. Rudolph, F. Rühr, A. Ruiz-Martinez, Z. Rurikova, N. A. Rusakovich, A. Ruschke, H. L. Russell, J. P. Rutherfoord, N. Ruthmann, Y. F. Ryabov, M. Rybar, G. Rybkin, S. Ryu, A. Ryzhov, G. F. Rzehorz, A. F. Saavedra, G. Sabato, S. Sacerdoti, H. F-W. Sadrozinski, R. Sadykov, F. Safai Tehrani, P. Saha, M. Sahinsoy, M. Saimpert, T. Saito, H. Sakamoto, Y. Sakurai, G. Salamanna, A. Salamon, J. E. Salazar Loyola, D. Salek, P. H. Sales De Bruin, D. Salihagic, A. Salnikov, J. Salt, D. Salvatore, F. Salvatore, A. Salvucci, A. Salzburger, D. Sammel, D. Sampsonidis, J. Sánchez, V. Sanchez Martinez, A. Sanchez Pineda, H. Sandaker, R. L. Sandbach, M. Sandhoff, C. Sandoval, D. P. C. Sankey, M. Sannino, A. Sansoni, C. Santoni, R. Santonico, H. Santos, I. Santoyo Castillo, K. Sapp, A. Sapronov, J. G. Saraiva, B. Sarrazin, O. Sasaki, K. Sato, E. Sauvan, G. Savage, P. Savard, N. Savic, C. Sawyer, L. Sawyer, J. Saxon, C. Sbarra, A. Sbrizzi, T. Scanlon, D. A. Scannicchio, M. Scarcella, V. Scarfone, J. Schaarschmidt, P. Schacht, B. M. Schachtner, D. Schaefer, L. Schaefer, R. Schaefer, J. Schaeffer, S. Schaepe, S. Schaetzel, U. Schäfer, A. C. Schaffer, D. Schaile, R. D. Schamberger, V. Scharf, V. A. Schegelsky, D. Scheirich, M. Schernau, C. Schiavi, S. Schier, C. Schillo, M. Schioppa, S. Schlenker, K. R. Schmidt-Sommerfeld, K. Schmieden, C. Schmitt, S. Schmitt, S. Schmitz, B. Schneider, U. Schnoor, L. Schoeffel, A. Schoening, B. D. Schoenrock, E. Schopf, M. Schott, J. F. P. Schouwenberg, J. Schovancova, S. Schramm, M. Schreyer, N. Schuh, A. Schulte, M. J. Schultens, H.-C. Schultz-Coulon, H. Schulz, M. Schumacher, B. A. Schumm, Ph. Schune, A. Schwartzman, T. A. Schwarz, H. Schweiger, Ph. Schwemling, R. Schwienhorst, J. Schwindling, T. Schwindt, G. Sciolla, F. Scuri, F. Scutti, J. Searcy, P. Seema, S. C. Seidel, A. Seiden, F. Seifert, J. M. Seixas, G. Sekhniaidze, K. Sekhon, S. J. Sekula, D. M. Seliverstov, N. Semprini-Cesari, C. Serfon, L. Serin, L. Serkin, M. Sessa, R. Seuster, H. Severini, T. Sfiligoj, F. Sforza, A. Sfyrla, E. Shabalina, N. W. Shaikh, L. Y. Shan, R. Shang, J. T. Shank, M. Shapiro, P. B. Shatalov, K. Shaw, S. M. Shaw, A. Shcherbakova, C. Y. Shehu, P. Sherwood, L. Shi, S. Shimizu, C. O. Shimmin, M. Shimojima, S. Shirabe, M. Shiyakova, A. Shmeleva, D. Shoaleh Saadi, M. J. Shochet, S. Shojaii, D. R. Shope, S. Shrestha, E. Shulga, M. A. Shupe, P. Sicho, A. M. Sickles, P. E. Sidebo, E. Sideras Haddad, O. Sidiropoulou, D. Sidorov, A. Sidoti, F. Siegert, Dj. Sijacki, J. Silva, S. B. Silverstein, V. Simak, Lj. Simic, S. Simion, E. Simioni, B. Simmons, D. Simon, M. Simon, P. Sinervo, N. B. Sinev, M. Sioli, G. Siragusa, S. Yu. Sivoklokov, J. Sjölin, M. B. Skinner, H. P. Skottowe, P. Skubic, M. Slater, T. Slavicek, M. Slawinska, K. Sliwa, R. Slovak, V. Smakhtin, B. H. Smart, L. Smestad, J. Smiesko, S. Yu. Smirnov, Y. Smirnov, L. N. Smirnova, O. Smirnova, M. N. K. Smith, R. W. Smith, M. Smizanska, K. Smolek, A. A. Snesarev, I. M. Snyder, S. Snyder, R. Sobie, F. Socher, A. Soffer, D. A. Soh, G. Sokhrannyi, C. A. Solans Sanchez, M. Solar, E. Yu. Soldatov, U. Soldevila, A. A. Solodkov, A. Soloshenko, O. V. Solovyanov, V. Solovyev, P. Sommer, H. Son, H. Y. Song, A. Sood, A. Sopczak, V. Sopko, V. Sorin, D. Sosa, C. L. Sotiropoulou, R. Soualah, A. M. Soukharev, D. South, B. C. Sowden, S. Spagnolo, M. Spalla, M. Spangenberg, F. Spanò, D. Sperlich, F. Spettel, R. Spighi, G. Spigo, L. A. Spiller, M. Spousta, R. D. St. Denis, A. Stabile, R. Stamen, S. Stamm, E. Stanecka, R. W. Stanek, C. Stanescu, M. Stanescu-Bellu, M. M. Stanitzki, S. Stapnes, E. A. Starchenko, G. H. Stark, J. Stark, P. Staroba, P. Starovoitov, S. Stärz, R. Staszewski, P. Steinberg, B. Stelzer, H. J. Stelzer, O. Stelzer-Chilton, H. Stenzel, G. A. Stewart, J. A. Stillings, M. C. Stockton, M. Stoebe, G. Stoicea, P. Stolte, S. Stonjek, A. R. Stradling, A. Straessner, M. E. Stramaglia, J. Strandberg, S. Strandberg, A. Strandlie, M. Strauss, P. Strizenec, R. Ströhmer, D. M. Strom, R. Stroynowski, A. Strubig, S. A. Stucci, B. Stugu, N. A. Styles, D. Su, J. Su, S. Suchek, Y. Sugaya, M. Suk, V. V. Sulin, S. Sultansoy, T. Sumida, S. Sun, X. Sun, J. E. Sundermann, K. Suruliz, G. Susinno, M. R. Sutton, S. Suzuki, M. Svatos, M. Swiatlowski, I. Sykora, T. Sykora, D. Ta, C. Taccini, K. Tackmann, J. Taenzer, A. Taffard, R. Tafirout, N. Taiblum, H. Takai, R. Takashima, T. Takeshita, Y. Takubo, M. Talby, A. A. Talyshev, K. G. Tan, J. Tanaka, M. Tanaka, R. Tanaka, S. Tanaka, R. Tanioka, B. B. Tannenwald, S. Tapia Araya, S. Tapprogge, S. Tarem, G. F. Tartarelli, P. Tas, M. Tasevsky, T. Tashiro, E. Tassi, A. Tavares Delgado, Y. Tayalati, A. C. Taylor, G. N. Taylor, P. T. E. Taylor, W. Taylor, F. A. Teischinger, P. Teixeira-Dias, K. K. Temming, D. Temple, H. Ten Kate, P. K. Teng, J. J. Teoh, F. Tepel, S. Terada, K. Terashi, J. Terron, S. Terzo, M. Testa, R. J. Teuscher, T. Theveneaux-Pelzer, J. P. Thomas, J. Thomas-Wilsker, P. D. Thompson, A. S. Thompson, L. A. Thomsen, E. Thomson, M. J. Tibbetts, R. E. Ticse Torres, V. O. Tikhomirov, Yu. A. Tikhonov, S. Timoshenko, P. Tipton, S. Tisserant, K. Todome, T. Todorov, S. Todorova-Nova, J. Tojo, S. Tokár, K. Tokushuku, E. Tolley, L. Tomlinson, M. Tomoto, L. Tompkins, K. Toms, B. Tong, P. Tornambe, E. Torrence, H. Torres, E. Torró Pastor, J. Toth, F. Touchard, D. R. Tovey, T. Trefzger, A. Tricoli, I. M. Trigger, S. Trincaz-Duvoid, M. F. Tripiana, W. Trischuk, B. Trocmé, A. Trofymov, C. Troncon, M. Trottier-McDonald, M. Trovatelli, L. Truong, M. Trzebinski, A. Trzupek, J. C-L. Tseng, P. V. Tsiareshka, G. Tsipolitis, N. Tsirintanis, S. Tsiskaridze, V. Tsiskaridze, E. G. Tskhadadze, K. M. Tsui, I. I. Tsukerman, V. Tsulaia, S. Tsuno, D. Tsybychev, Y. Tu, A. Tudorache, V. Tudorache, A. N. Tuna, S. A. Tupputi, S. Turchikhin, D. Turecek, D. Turgeman, R. Turra, P. M. Tuts, M. Tyndel, G. Ucchielli, I. Ueda, M. Ughetto, F. Ukegawa, G. Unal, A. Undrus, G. Unel, F. C. Ungaro, Y. Unno, C. Unverdorben, J. Urban, P. Urquijo, P. Urrejola, G. Usai, J. Usui, L. Vacavant, V. Vacek, B. Vachon, C. Valderanis, E. Valdes Santurio, N. Valencic, S. Valentinetti, A. Valero, L. Valery, S. Valkar, J. A. Valls Ferrer, W. Van Den Wollenberg, P. C. Van Der Deijl, H. van der Graaf, N. van Eldik, P. van Gemmeren, J. Van Nieuwkoop, I. van Vulpen, M. C. van Woerden, M. Vanadia, W. Vandelli, R. Vanguri, A. Vaniachine, P. Vankov, G. Vardanyan, R. Vari, E. W. Varnes, T. Varol, D. Varouchas, A. Vartapetian, K. E. Varvell, J. G. Vasquez, G. A. Vasquez, F. Vazeille, T. Vazquez Schroeder, J. Veatch, V. Veeraraghavan, L. M. Veloce, F. Veloso, S. Veneziano, A. Ventura, M. Venturi, N. Venturi, A. Venturini, V. Vercesi, M. Verducci, W. Verkerke, J. C. Vermeulen, A. Vest, M. C. Vetterli, O. Viazlo, I. Vichou, T. Vickey, O. E. Vickey Boeriu, G. H. A. Viehhauser, S. Viel, L. Vigani, M. Villa, M. Villaplana Perez, E. Vilucchi, M. G. Vincter, V. B. Vinogradov, C. Vittori, I. Vivarelli, S. Vlachos, M. Vlasak, M. Vogel, P. Vokac, G. Volpi, M. Volpi, H. von der Schmitt, E. von Toerne, V. Vorobel, K. Vorobev, M. Vos, R. Voss, J. H. Vossebeld, N. Vranjes, M. Vranjes Milosavljevic, V. Vrba, M. Vreeswijk, R. Vuillermet, I. Vukotic, Z. Vykydal, P. Wagner, W. Wagner, H. Wahlberg, S. Wahrmund, J. Wakabayashi, J. Walder, R. Walker, W. Walkowiak, V. Wallangen, C. Wang, C. Wang, F. Wang, H. Wang, H. Wang, J. Wang, J. Wang, K. Wang, R. Wang, S. M. Wang, T. Wang, T. Wang, W. Wang, C. Wanotayaroj, A. Warburton, C. P. Ward, D. R. Wardrope, A. Washbrook, P. M. Watkins, A. T. Watson, M. F. Watson, G. Watts, S. Watts, B. M. Waugh, S. Webb, M. S. Weber, S. W. Weber, S. A. Weber, J. S. Webster, A. R. Weidberg, B. Weinert, J. Weingarten, C. Weiser, H. Weits, P. S. Wells, T. Wenaus, T. Wengler, S. Wenig, N. Wermes, M. Werner, M. D. Werner, P. Werner, M. Wessels, J. Wetter, K. Whalen, N. L. Whallon, A. M. Wharton, A. White, M. J. White, R. White, D. Whiteson, F. J. Wickens, W. Wiedenmann, M. Wielers, C. Wiglesworth, L. A. M. Wiik-Fuchs, A. Wildauer, F. Wilk, H. G. Wilkens, H. H. Williams, S. Williams, C. Willis, S. Willocq, J. A. Wilson, I. Wingerter-Seez, F. Winklmeier, O. J. Winston, B. T. Winter, M. Wittgen, J. Wittkowski, T. M. H. Wolf, M. W. Wolter, H. Wolters, S. D. Worm, B. K. Wosiek, J. Wotschack, M. J. Woudstra, K. W. Wozniak, M. Wu, M. Wu, S. L. Wu, X. Wu, Y. Wu, T. R. Wyatt, B. M. Wynne, S. Xella, D. Xu, L. Xu, B. Yabsley, S. Yacoob, D. Yamaguchi, Y. Yamaguchi, A. Yamamoto, S. Yamamoto, T. Yamanaka, K. Yamauchi, Y. Yamazaki, Z. Yan, H. Yang, H. Yang, Y. Yang, Z. Yang, W-M. Yao, Y. C. Yap, Y. Yasu, E. Yatsenko, K. H. Yau Wong, J. Ye, S. Ye, I. Yeletskikh, E. Yildirim, K. Yorita, R. Yoshida, K. Yoshihara, C. Young, C. J. S. Young, S. Youssef, D. R. Yu, J. Yu, J. M. Yu, J. Yu, L. Yuan, S. P. Y. Yuen, I. Yusuff, B. Zabinski, R. Zaidan, A. M. Zaitsev, N. Zakharchuk, J. Zalieckas, A. Zaman, S. Zambito, L. Zanello, D. Zanzi, C. Zeitnitz, M. Zeman, A. Zemla, J. C. Zeng, Q. Zeng, O. Zenin, T. Ženiš, D. Zerwas, D. Zhang, F. Zhang, G. Zhang, H. Zhang, J. Zhang, L. Zhang, M. Zhang, R. Zhang, R. Zhang, X. Zhang, Z. Zhang, X. Zhao, Y. Zhao, Z. Zhao, A. Zhemchugov, J. Zhong, B. Zhou, C. Zhou, L. Zhou, L. Zhou, M. Zhou, N. Zhou, C. G. Zhu, H. Zhu, J. Zhu, Y. Zhu, X. Zhuang, K. Zhukov, A. Zibell, D. Zieminska, N. I. Zimine, C. Zimmermann, S. Zimmermann, Z. Zinonos, M. Zinser, M. Ziolkowski, L. Živković, G. Zobernig, A. Zoccoli, M. zur Nedden, L. Zwalinski, ATLAS Collaboration

**Affiliations:** 1 Department of Physics, University of Adelaide, Adelaide, Australia; 2 Physics Department, SUNY Albany, Albany, NY USA; 3 Department of Physics, University of Alberta, Edmonton, AB Canada; 4 Department of Physics, Ankara University, Ankara, Turkey; 5 Istanbul Aydin University, Istanbul, Turkey; 6 Division of Physics, TOBB University of Economics and Technology, Ankara, Turkey; 7 LAPP, CNRS/IN2P3 and Université Savoie Mont Blanc, Annecy-le-Vieux, France; 8 High Energy Physics Division, Argonne National Laboratory, Argonne, IL USA; 9 Department of Physics, University of Arizona, Tucson, AZ USA; 10 Department of Physics, The University of Texas at Arlington, Arlington, TX USA; 11 Physics Department, National and Kapodistrian University of Athens, Athens, Greece; 12 Physics Department, National Technical University of Athens, Zografou, Greece; 13 Department of Physics, The University of Texas at Austin, Austin, TX USA; 14 Institute of Physics, Azerbaijan Academy of Sciences, Baku, Azerbaijan; 15 Institut de Física d’Altes Energies (IFAE), The Barcelona Institute of Science and Technology, Barcelona, Spain; 16 Institute of Physics, University of Belgrade, Belgrade, Serbia; 17 Department for Physics and Technology, University of Bergen, Bergen, Norway; 18 Physics Division, Lawrence Berkeley National Laboratory and University of California, Berkeley, CA USA; 19 Department of Physics, Humboldt University, Berlin, Germany; 20 Albert Einstein Center for Fundamental Physics and Laboratory for High Energy Physics, University of Bern, Bern, Switzerland; 21 School of Physics and Astronomy, University of Birmingham, Birmingham, UK; 22 Department of Physics, Bogazici University, Istanbul, Turkey; 23 Department of Physics Engineering, Gaziantep University, Gaziantep, Turkey; 24 Faculty of Engineering and Natural Sciences, Istanbul Bilgi University, Istanbul, Turkey; 25 Faculty of Engineering and Natural Sciences, Bahcesehir University, Istanbul, Turkey; 26 Centro de Investigaciones, Universidad Antonio Narino, Bogotá, Colombia; 27 INFN Sezione di Bologna, Bologna, Italy; 28 Dipartimento di Fisica e Astronomia, Università di Bologna, Bologna, Italy; 29 Physikalisches Institut, University of Bonn, Bonn, Germany; 30 Department of Physics, Boston University, Boston, MA USA; 31 Department of Physics, Brandeis University, Waltham, MA USA; 32 Universidade Federal do Rio De Janeiro COPPE/EE/IF, Rio de Janeiro, Brazil; 33 Electrical Circuits Department, Federal University of Juiz de Fora (UFJF), Juiz de Fora, Brazil; 34 Federal University of Sao Joao del Rei (UFSJ), Sao Joao del Rei, Brazil; 35 Instituto de Fisica, Universidade de Sao Paulo, Sao Paulo, Brazil; 36 Physics Department, Brookhaven National Laboratory, Upton, NY USA; 37 Transilvania University of Brasov, Brasov, Romania; 38 National Institute of Physics and Nuclear Engineering, Bucharest, Romania; 39 Physics Department, National Institute for Research and Development of Isotopic and Molecular Technologies, Cluj-Napoca, Romania; 40 University Politehnica Bucharest, Bucharest, Romania; 41 West University in Timisoara, Timisoara, Romania; 42 Departamento de Física, Universidad de Buenos Aires, Buenos Aires, Argentina; 43 Cavendish Laboratory, University of Cambridge, Cambridge, UK; 44 Department of Physics, Carleton University, Ottawa, ON Canada; 45 CERN, Geneva, Switzerland; 46 Enrico Fermi Institute, University of Chicago, Chicago, IL USA; 47 Departamento de Física, Pontificia Universidad Católica de Chile, Santiago, Chile; 48 Departamento de Física, Universidad Técnica Federico Santa María, Valparaiso, Chile; 49 Institute of High Energy Physics, Chinese Academy of Sciences, Beijing, China; 50 Department of Physics, Nanjing University, Jiangsu, China; 51 Physics Department, Tsinghua University, Beijing, 100084 China; 52 Laboratoire de Physique Corpusculaire, Université Clermont Auvergne, Université Blaise Pascal, CNRS/IN2P3, Clermont-Ferrand, France; 53 Nevis Laboratory, Columbia University, Irvington, NY USA; 54 Niels Bohr Institute, University of Copenhagen, Kobenhavn, Denmark; 55 INFN Gruppo Collegato di Cosenza, Laboratori Nazionali di Frascati, Frascati, Italy; 56 Dipartimento di Fisica, Università della Calabria, Rende, Italy; 57 Faculty of Physics and Applied Computer Science, AGH University of Science and Technology, Kraków, Poland; 58 Marian Smoluchowski Institute of Physics, Jagiellonian University, Kraków, Poland; 59 Institute of Nuclear Physics Polish Academy of Sciences, Kraków, Poland; 60 Physics Department, Southern Methodist University, Dallas, TX USA; 61 Physics Department, University of Texas at Dallas, Richardson, TX USA; 62 DESY, Hamburg and Zeuthen, Germany; 63 Lehrstuhl für Experimentelle Physik IV, Technische Universität Dortmund, Dortmund, Germany; 64 Institut für Kern- und Teilchenphysik, Technische Universität Dresden, Dresden, Germany; 65 Department of Physics, Duke University, Durham, NC USA; 66 SUPA-School of Physics and Astronomy, University of Edinburgh, Edinburgh, UK; 67 INFN Laboratori Nazionali di Frascati, Frascati, Italy; 68 Fakultät für Mathematik und Physik, Albert-Ludwigs-Universität, Freiburg, Germany; 69 Departement de Physique Nucleaire et Corpusculaire, Université de Genève, Geneva, Switzerland; 70 INFN Sezione di Genova, Genoa, Italy; 71 Dipartimento di Fisica, Università di Genova, Genoa, Italy; 72 E. Andronikashvili Institute of Physics, Iv. Javakhishvili Tbilisi State University, Tbilisi, Georgia; 73 High Energy Physics Institute, Tbilisi State University, Tbilisi, Georgia; 74 II Physikalisches Institut, Justus-Liebig-Universität Giessen, Giessen, Germany; 75 SUPA-School of Physics and Astronomy, University of Glasgow, Glasgow, UK; 76 II Physikalisches Institut, Georg-August-Universität, Göttingen, Germany; 77 Laboratoire de Physique Subatomique et de Cosmologie, Université Grenoble-Alpes, CNRS/IN2P3, Grenoble, France; 78 Laboratory for Particle Physics and Cosmology, Harvard University, Cambridge, MA USA; 79 Department of Modern Physics, University of Science and Technology of China, Anhui, China; 80 Kirchhoff-Institut für Physik, Ruprecht-Karls-Universität Heidelberg, Heidelberg, Germany; 81 Physikalisches Institut, Ruprecht-Karls-Universität Heidelberg, Heidelberg, Germany; 82 ZITI Institut für technische Informatik, Ruprecht-Karls-Universität Heidelberg, Mannheim, Germany; 83 Faculty of Applied Information Science, Hiroshima Institute of Technology, Hiroshima, Japan; 84 Department of Physics, The Chinese University of Hong Kong, Shatin, N.T. Hong Kong; 85 Department of Physics, The University of Hong Kong, Hong Kong, China; 86 Department of Physics and Institute for Advanced Study, The Hong Kong University of Science and Technology, Clear Water Bay, Kowloon, Hong Kong, China; 87 Department of Physics, Indiana University, Bloomington, IN USA; 88 Institut für Astro- und Teilchenphysik, Leopold-Franzens-Universität, Innsbruck, Austria; 89 University of Iowa, Iowa City, IA USA; 90 Department of Physics and Astronomy, Iowa State University, Ames, IA USA; 91 Joint Institute for Nuclear Research, JINR Dubna, Dubna, Russia; 92 KEK, High Energy Accelerator Research Organization, Tsukuba, Japan; 93 Graduate School of Science, Kobe University, Kobe, Japan; 94 Faculty of Science, Kyoto University, Kyoto, Japan; 95 Kyoto University of Education, Kyoto, Japan; 96 Department of Physics, Kyushu University, Fukuoka, Japan; 97 Instituto de Física La Plata, Universidad Nacional de La Plata and CONICET, La Plata, Argentina; 98 Physics Department, Lancaster University, Lancaster, UK; 99 INFN Sezione di Lecce, Lecce, Italy; 100 Dipartimento di Matematica e Fisica, Università del Salento, Lecce, Italy; 101 Oliver Lodge Laboratory, University of Liverpool, Liverpool, UK; 102 Department of Experimental Particle Physics, Jožef Stefan Institute and Department of Physics, University of Ljubljana, Ljubljana, Slovenia; 103 School of Physics and Astronomy, Queen Mary University of London, London, UK; 104 Department of Physics, Royal Holloway University of London, Surrey, UK; 105 Department of Physics and Astronomy, University College London, London, UK; 106 Louisiana Tech University, Ruston, LA USA; 107 Laboratoire de Physique Nucléaire et de Hautes Energies, UPMC and Université Paris-Diderot and CNRS/IN2P3, Paris, France; 108 Fysiska institutionen, Lunds universitet, Lund, Sweden; 109 Departamento de Fisica Teorica C-15, Universidad Autonoma de Madrid, Madrid, Spain; 110 Institut für Physik, Universität Mainz, Mainz, Germany; 111 School of Physics and Astronomy, University of Manchester, Manchester, UK; 112 CPPM, Aix-Marseille Université and CNRS/IN2P3, Marseille, France; 113 Department of Physics, University of Massachusetts, Amherst, MA USA; 114 Department of Physics, McGill University, Montreal, QC Canada; 115 School of Physics, University of Melbourne, Victoria, Australia; 116 Department of Physics, The University of Michigan, Ann Arbor, MI USA; 117 Department of Physics and Astronomy, Michigan State University, East Lansing, MI USA; 118 INFN Sezione di Milano, Milan, Italy; 119 Dipartimento di Fisica, Università di Milano, Milan, Italy; 120 B.I. Stepanov Institute of Physics, National Academy of Sciences of Belarus, Minsk, Republic of Belarus; 121 Research Institute for Nuclear Problems of Byelorussian State University, Minsk, Republic of Belarus; 122 Group of Particle Physics, University of Montreal, Montreal, QC Canada; 123 P.N. Lebedev Physical Institute of the Russian Academy of Sciences, Moscow, Russia; 124 Institute for Theoretical and Experimental Physics (ITEP), Moscow, Russia; 125 National Research Nuclear University MEPhI, Moscow, Russia; 126 D.V. Skobeltsyn Institute of Nuclear Physics, M.V. Lomonosov Moscow State University, Moscow, Russia; 127 Fakultät für Physik, Ludwig-Maximilians-Universität München, Munich, Germany; 128 Max-Planck-Institut für Physik (Werner-Heisenberg-Institut), München, Germany; 129 Nagasaki Institute of Applied Science, Nagasaki, Japan; 130 Graduate School of Science and Kobayashi-Maskawa Institute, Nagoya University, Nagoya, Japan; 131 INFN Sezione di Napoli, Napoli, Italy; 132 Dipartimento di Fisica, Università di Napoli, Napoli, Italy; 133 Department of Physics and Astronomy, University of New Mexico, Albuquerque, NM USA; 134 Institute for Mathematics, Astrophysics and Particle Physics, Radboud University Nijmegen/Nikhef, Nijmegen, The Netherlands; 135 Nikhef National Institute for Subatomic Physics and University of Amsterdam, Amsterdam, The Netherlands; 136 Department of Physics, Northern Illinois University, DeKalb, IL USA; 137 Budker Institute of Nuclear Physics, SB RAS, Novosibirsk, Russia; 138 Department of Physics, New York University, New York, NY USA; 139 Ohio State University, Columbus, OH USA; 140 Faculty of Science, Okayama University, Okayama, Japan; 141 Homer L. Dodge Department of Physics and Astronomy, University of Oklahoma, Norman, OK USA; 142 Department of Physics, Oklahoma State University, Stillwater, OK USA; 143 Palacký University, RCPTM, Olomouc, Czech Republic; 144 Center for High Energy Physics, University of Oregon, Eugene, OR USA; 145 LAL, Univ. Paris-Sud, CNRS/IN2P3, Université Paris-Saclay, Orsay, France; 146 Graduate School of Science, Osaka University, Osaka, Japan; 147 Department of Physics, University of Oslo, Oslo, Norway; 148 Department of Physics, Oxford University, Oxford, UK; 149 INFN Sezione di Pavia, Pavia, Italy; 150 Dipartimento di Fisica, Università di Pavia, Pavia, Italy; 151 Department of Physics, University of Pennsylvania, Philadelphia, PA USA; 152 National Research Centre “Kurchatov Institute” B.P. Konstantinov Petersburg Nuclear Physics Institute, St. Petersburg, Russia; 153 INFN Sezione di Pisa, Pisa, Italy; 154 Dipartimento di Fisica E. Fermi, Università di Pisa, Pisa, Italy; 155 Department of Physics and Astronomy, University of Pittsburgh, Pittsburgh, PA USA; 156 Laboratório de Instrumentação e Física Experimental de Partículas-LIP, Lisbon, Portugal; 157 Faculdade de Ciências, Universidade de Lisboa, Lisbon, Portugal; 158 Department of Physics, University of Coimbra, Coimbra, Portugal; 159 Centro de Física Nuclear da Universidade de Lisboa, Lisbon, Portugal; 160 Departamento de Fisica, Universidade do Minho, Braga, Portugal; 161 Departamento de Fisica Teorica y del Cosmos and CAFPE, Universidad de Granada, Granada, Spain; 162 Dep Fisica and CEFITEC of Faculdade de Ciencias e Tecnologia, Universidade Nova de Lisboa, Caparica, Portugal; 163 Institute of Physics, Academy of Sciences of the Czech Republic, Praha, Czech Republic; 164 Czech Technical University in Prague, Praha, Czech Republic; 165 Faculty of Mathematics and Physics, Charles University in Prague, Praha, Czech Republic; 166 State Research Center Institute for High Energy Physics (Protvino), NRC KI, Protvino, Russia; 167 Particle Physics Department, Rutherford Appleton Laboratory, Didcot, UK; 168 INFN Sezione di Roma, Rome, Italy; 169 Dipartimento di Fisica, Sapienza Università di Roma, Rome, Italy; 170 INFN Sezione di Roma Tor Vergata, Rome, Italy; 171 Dipartimento di Fisica, Università di Roma Tor Vergata, Rome, Italy; 172 INFN Sezione di Roma Tre, Rome, Italy; 173 Dipartimento di Matematica e Fisica, Università Roma Tre, Rome, Italy; 174 Faculté des Sciences Ain Chock, Réseau Universitaire de Physique des Hautes Energies-Université Hassan II, Casablanca, Morocco; 175 Centre National de l’Energie des Sciences Techniques Nucleaires, Rabat, Morocco; 176 Faculté des Sciences Semlalia, Université Cadi Ayyad, LPHEA-Marrakech, Marrakech, Morocco; 177 Faculté des Sciences, Université Mohamed Premier and LPTPM, Oujda, Morocco; 178 Faculté des Sciences, Université Mohammed V, Rabat, Morocco; 179 DSM/IRFU (Institut de Recherches sur les Lois Fondamentales de l’Univers), CEA Saclay (Commissariat à l’Energie Atomique et aux Energies Alternatives), Gif-sur-Yvette, France; 180 Santa Cruz Institute for Particle Physics, University of California Santa Cruz, Santa Cruz, CA USA; 181 Department of Physics, University of Washington, Seattle, WA USA; 182 School of Physics, Shandong University, Shandong, China; 183 Department of Physics and Astronomy, Shanghai Key Laboratory for Particle Physics and Cosmology, Shanghai Jiao Tong University; (Also Affiliated with PKU-CHEP), Shanghai, China; 184 Department of Physics and Astronomy, University of Sheffield, Sheffield, UK; 185 Department of Physics, Shinshu University, Nagano, Japan; 186 Fachbereich Physik, Universität Siegen, Siegen, Germany; 187 Department of Physics, Simon Fraser University, Burnaby, BC Canada; 188 SLAC National Accelerator Laboratory, Stanford, CA USA; 189 Faculty of Mathematics, Physics and Informatics, Comenius University, Bratislava, Slovak Republic; 190 Department of Subnuclear Physics, Institute of Experimental Physics of the Slovak Academy of Sciences, Kosice, Slovak Republic; 191 Department of Physics, University of Cape Town, Cape Town, South Africa; 192 Department of Physics, University of Johannesburg, Johannesburg, South Africa; 193 School of Physics, University of the Witwatersrand, Johannesburg, South Africa; 194 Department of Physics, Stockholm University, Stockholm, Sweden; 195 The Oskar Klein Centre, Stockholm, Sweden; 196 Physics Department, Royal Institute of Technology, Stockholm, Sweden; 197 Departments of Physics and Astronomy and Chemistry, Stony Brook University, Stony Brook, NY USA; 198 Department of Physics and Astronomy, University of Sussex, Brighton, UK; 199 School of Physics, University of Sydney, Sydney, Australia; 200 Institute of Physics, Academia Sinica, Taipei, Taiwan; 201 Department of Physics, Technion: Israel Institute of Technology, Haifa, Israel; 202 Raymond and Beverly Sackler School of Physics and Astronomy, Tel Aviv University, Tel Aviv, Israel; 203 Department of Physics, Aristotle University of Thessaloniki, Thessaloniki, Greece; 204 International Center for Elementary Particle Physics and Department of Physics, The University of Tokyo, Tokyo, Japan; 205 Graduate School of Science and Technology, Tokyo Metropolitan University, Tokyo, Japan; 206 Department of Physics, Tokyo Institute of Technology, Tokyo, Japan; 207 Tomsk State University, Tomsk, Russia; 208 Department of Physics, University of Toronto, Toronto, ON Canada; 209 INFN-TIFPA, Trento, Italy; 210 University of Trento, Trento, Italy; 211 TRIUMF, Vancouver, BC Canada; 212 Department of Physics and Astronomy, York University, Toronto, ON Canada; 213 Faculty of Pure and Applied Sciences, and Center for Integrated Research in Fundamental Science and Engineering, University of Tsukuba, Tsukuba, Japan; 214 Department of Physics and Astronomy, Tufts University, Medford, MA USA; 215 Department of Physics and Astronomy, University of California Irvine, Irvine, CA USA; 216 INFN Gruppo Collegato di Udine, Sezione di Trieste, Udine, Italy; 217 ICTP, Trieste, Italy; 218 Dipartimento di Chimica, Fisica e Ambiente, Università di Udine, Udine, Italy; 219 Department of Physics and Astronomy, University of Uppsala, Uppsala, Sweden; 220 Department of Physics, University of Illinois, Urbana, IL USA; 221 Instituto de Fisica Corpuscular (IFIC) and Departamento de Fisica Atomica, Molecular y Nuclear and Departamento de Ingeniería Electrónica and Instituto de Microelectrónica de Barcelona (IMB-CNM), University of Valencia and CSIC, Valencia, Spain; 222 Department of Physics, University of British Columbia, Vancouver, BC Canada; 223 Department of Physics and Astronomy, University of Victoria, Victoria, BC Canada; 224 Department of Physics, University of Warwick, Coventry, UK; 225 Waseda University, Tokyo, Japan; 226 Department of Particle Physics, The Weizmann Institute of Science, Rehovot, Israel; 227 Department of Physics, University of Wisconsin, Madison, WI USA; 228 Fakultät für Physik und Astronomie, Julius-Maximilians-Universität, Würzburg, Germany; 229 Fakultät für Mathematik und Naturwissenschaften, Fachgruppe Physik, Bergische Universität Wuppertal, Wuppertal, Germany; 230 Department of Physics, Yale University, New Haven, CT USA; 231 Yerevan Physics Institute, Yerevan, Armenia; 232 Centre de Calcul de l’Institut National de Physique Nucléaire et de Physique des Particules (IN2P3), Villeurbanne, France; 233 CERN, 1211 Geneva 23, Switzerland

## Abstract

This article presents measurements of $t\bar{t}$ differential cross-sections in a fiducial phase-space region, using an integrated luminosity of 3.2 fb$^{-1}$ of proton–proton data at a centre-of-mass energy of $\sqrt{s} = 13$ TeV recorded by the ATLAS experiment at the LHC in 2015. Differential cross-sections are measured as a function of the transverse momentum and absolute rapidity of the top quark, and of the transverse momentum, absolute rapidity and invariant mass of the $t\bar{t}$ system. The $t\bar{t}$ events are selected by requiring one electron and one muon of opposite electric charge, and at least two jets, one of which must be tagged as containing a *b*-hadron. The measured differential cross-sections are compared to predictions of next-to-leading order generators matched to parton showers and the measurements are found to be consistent with all models within the experimental uncertainties with the exception of the Powheg-Box $+$ Herwig++ predictions, which differ significantly from the data in both the transverse momentum of the top quark and the mass of the $t\bar{t}$ system.

## Introduction

The top quark is the heaviest fundamental particle in the standard model (SM) of particle physics. Understanding the production cross-section and kinematics of $t\bar{t}$ pairs is an important test of SM predictions. Furthermore, $t\bar{t}$ production is often an important background in searches for new physics and a detailed understanding of this process is therefore crucial.

At the large hadron collider (LHC), $t\bar{t}$ pair production in proton–proton (*pp*) collisions at a centre-of-mass energy of $\sqrt{s}=13$ TeV occurs predominantly via gluon fusion (90%) with small contributions from $q\bar{q}$ annihilation (10%). Significant progress has been made in the precision of the calculations of the cross-section of this process, both inclusive and differential. Currently, calculations are available at next-to-next-to-leading order (NNLO) in perturbative QCD, including the resummation of next-to-next-to-leading logarithmic (NNLL) soft gluon terms [1–11].

Differential cross-sections for $t\bar{t}$ production have been measured by the ATLAS [12–14] and CMS [15, 16] experiments, in events containing either one or two charged leptons, at $\sqrt{s}=7$ TeV and $\sqrt{s}=8$ TeV. Measurements of $t\bar{t}$ differential cross-sections at $\sqrt{s}=13$ TeV have also been made at the CMS experiment [17] in events containing one charged lepton. The integrated luminosity of 3.2 fb$^{-1}$ of *pp* collision data collected by the ATLAS experiment at $\sqrt{s}=13$ TeV allows the measurement of the differential cross-section as a function of the kinematic variables of the $t\bar{t}$ system in a different kinematic regime compared to the previous LHC measurements. The inclusive cross-section has been measured at $\sqrt{s}=13$ TeV by both the ATLAS [18] and CMS [19, 20] experiments and was found to be in agreement with the theoretical predictions. This article presents measurements of $t\bar{t}$ differential cross-sections in terms of five different kinematic observables, both absolute and normalised to the fiducial cross-section. These observables are the transverse momentum of the top quark ($p_{\text{ T }}(t)$), the absolute rapidity of the top quark (|*y*(*t*)|), the transverse momentum of the $t\bar{t}$ system ($p_{\text{ T }}(t\bar{t})$), the absolute rapidity of the $t\bar{t}$ system ($|y(t\bar{t})|$), and the invariant mass of the $t\bar{t}$ system ($m(t\bar{t})$). The distributions of these variables are unfolded to the particle level in a fiducial volume. The $p_{\text{ T }}(t)$ and $m(t\bar{t})$ observables are expected to be sensitive to the modelling of higher-order corrections in QCD, whereas the rapidity of the top quark and $t\bar{t}$ system are expected to have sensitivity to the parton distribution functions (PDF) used in the simulations. The $p_{\text{ T }}(t\bar{t})$ observable is sensitive to the amount of gluon radiation in the event and can be useful for the tuning of Monte Carlo (MC) generators. Top quarks and anti-top quarks are measured in one combined distribution for the $p_{\text{ T }}(t)$ and |*y*(*t*)| observables, rather than studying them separately. The $t\bar{t}$ system is reconstructed in events containing exactly one electron and one muon. Events in which a $\tau$ lepton decays to an electron or muon are also included.

## ATLAS detector

The ATLAS detector [21] at the LHC covers nearly the entire solid angle around the interaction point. It consists of an inner tracking detector surrounded by a thin superconducting solenoid, electromagnetic and hadronic calorimeters, and a muon spectrometer incorporating three large superconducting toroidal magnet systems. The inner-detector system is immersed in a 2 T axial magnetic field and provides charged-particle tracking in the range $|\eta | < 2.5$.^1^

The high-granularity silicon pixel detector surrounds the collision region and provides four measurements per track. The closest layer, known as the Insertable B-Layer [22, 23], was added in 2014 and provides high-resolution hits at small radius to improve the tracking performance. The pixel detector is followed by the silicon microstrip tracker, which provides four three-dimensional measurement points per track. These silicon detectors are complemented by the transition radiation tracker, which enables radially extended track reconstruction up to $|\eta | = 2.0$. The transition radiation tracker also provides electron identification information based on the fraction of hits (typically 30 in total) passing a higher charge threshold indicative of transition radiation.

The calorimeter system covers the pseudorapidity range $|\eta | < 4.9$. Within the region $|\eta |< 3.2$, electromagnetic calorimetry is provided by barrel and endcap high-granularity lead/liquid-argon (LAr) electromagnetic calorimeters, with an additional thin LAr presampler covering $|\eta | < 1.8$ to correct for energy loss in material upstream of the calorimeters. Hadronic calorimetry is provided by the steel/scintillator-tile calorimeter, segmented into three barrel structures within $|\eta | < 1.7$, and two copper/LAr hadronic endcap calorimeters that cover $1.5< |\eta | < 3.2$. The solid angle coverage is completed with forward copper/LAr and tungsten/LAr calorimeter modules optimised for electromagnetic and hadronic measurements respectively, in the region $3.1< |\eta | < 4.9$.

The muon spectrometer comprises separate trigger and high-precision tracking chambers measuring the deflection of muons in a magnetic field generated by superconducting air-core toroids. The precision chamber system covers the region $|\eta | < 2.7$ with three layers of monitored drift tubes, complemented by cathode strip chambers in the forward region, where the background is highest. The muon trigger system covers the range $|\eta | < 2.4$ with resistive-plate chambers in the barrel, and thin-gap chambers in the endcap regions.

A two-level trigger system is used to select interesting events [24, 25]. The Level-1 trigger is implemented in hardware and uses a subset of detector information to reduce the event rate to a design value of at most 100 kHz. This is followed by the software-based high-level trigger, which reduces the event rate to 1 kHz.

## Data and simulation samples

The *pp* collision data used in this analysis were collected during 2015 by ATLAS and correspond to an integrated luminosity of 3.2 fb$^{-1}$ at $\sqrt{s} = 13$ TeV. The data considered in this analysis were collected under stable beam conditions, and requiring all subdetectors to be operational. Each selected event includes additional interactions from, on average, 14 inelastic *pp* collisions in the same proton bunch crossing, as well as residual detector signals from previous bunch crossings with a 25 ns bunch spacing, collectively referred to as “pile-up”. Events are required to pass a single-lepton trigger, either electron or muon. Multiple triggers are used to select events: either triggers with low $p_{\text {T}}$ thresholds of 24 GeV that utilise isolation requirements to reduce the trigger rate, or higher $p_{\text {T}}$ thresholds of 50 GeV for muons or 60 and 120 GeV for electrons, with no isolation requirements to increase event acceptance.

MC simulations are used to model background processes and to correct the data for detector acceptance and resolution effects. The ATLAS detector is simulated [26] using Geant 4 [27]. A “fast simulation” [28], utilising parameterised showers in the calorimeter, but with full simulation of the inner detector and muon spectrometer, is used in the samples generated to estimate $t\bar{t}$ modelling uncertainties. Additional *pp* interactions are generated using Pythia 8 (v8.186) [29] and overlaid on signal and background processes in order to simulate the effect of pile-up. The MC simulations are reweighted to match the distribution of the average number of interactions per bunch crossing that are observed in data. This process is referred to as “pile-up reweighting”. The same reconstruction algorithms and analysis procedures are applied to both data and MC simulation. Corrections derived from dedicated data samples are applied to the MC simulation in order to improve agreement with data.

The nominal $t\bar{t}$ sample is simulated using the next-to-leading order (NLO) Powheg-Box (v2) matrix-element event generator [30–32] using Pythia 6 (v6.427) [33] for the parton shower (PS). Powheg-Box is interfaced to the CT10 [34] NLO PDF set while Pythia 6 uses the CTEQ6L1 PDF set [35]. A set of tuned parameters called the Perugia 2012 tune [36] is used in the simulation of the underlying event. The “$h_{\mathrm {damp}}$” parameter, which controls the $p_{\text {T}}$ of the first additional gluon emission beyond the Born configuration, is set to the mass of the top quark ($m_{t}$). The main effect of this is to regulate the high-$p_{\text {T}}$ emission against which the $t\bar{t}$ system recoils. The choice of this $h_{\mathrm {damp}}$ value was found to improve the modelling of the $t\bar{t}$ system kinematics with respect to data in previous analyses [37]. In order to investigate the effects of initial- and final-state radiation, alternative Powheg-Box $+$ Pythia 6 samples are generated with the renormalisation and factorisation scales varied by a factor of 2 (0.5) and using low (high) radiation variations of the Perugia 2012 tune and an $h_{\mathrm {damp}}$ value of $m_{t}$ ($2m_{t}$), corresponding to less (more) parton-shower radiation [37], referred to as “radHi” and “radLo”. These variations were selected to cover the uncertainties in the measurements of differential distributions in $\sqrt{s}=7$ TeV data [12]. The $h_{\mathrm {damp}}$ value for the low radiation sample is not decreased as it was found to disagree with previously published data. Alternative samples are generated using Powheg-Box (v2) and MadGraph5_aMC@NLO  (v2.2.1) [38], referred to as MG5_aMC@NLO hereafter, both interfaced to Herwig++  (v2.7.1) [39], in order to estimate the effects of the choice of matrix-element event generator and parton-shower algorithm. Additional $t\bar{t}$ samples are generated for comparisons with unfolded data using Sherpa  (v2.2.0) [40], Powheg-Box (v2) $+$ Pythia 8 as well as Powheg-Box (v2) and MG5_aMC@NLO interfaced to Herwig 7 [39, 41]. In all $t\bar{t}$ samples, the mass of the top quark is set to 172.5 GeV. These $t\bar{t}$ samples are described in further detail in Ref. [37].

Background processes are simulated using a variety of MC event generators. Single-top quark production in association with a *W* boson (*Wt*) is simulated using Powheg-Box v1 $+$ Pythia 6 with the same parameters and PDF sets as those used for the nominal $t\bar{t}$ sample and is normalised to the theoretical cross-section [42]. The higher-order overlap with $t\bar{t}$ production is addressed using the “diagram removal” (DR) generation scheme [43]. A sample generated using an alternative “diagram subtraction” (DS) method is used to evaluate systematic uncertainties [43].

Sherpa  (v2.1.1), interfaced to the CT10 PDF set, is used to model Drell–Yan production, where the dominant contribution is from $Z/\gamma ^*\rightarrow \tau ^+\tau ^-$. For this process, Sherpa calculates matrix elements at NLO for up to two partons and at leading order (LO) for up to four partons using the OpenLoops [44] and Comix [45] matrix-element event generators. The matrix elements are merged with the Sherpa parton shower [46] using the ME $+$ PS@NLO prescription [47]. The total cross-section is normalised to the NNLO predictions [48]. Sherpa  (v2.1.1) with the CT10 PDF set is also used to simulate electroweak diboson production [49] (*WW*, *WZ*, *ZZ*), where both bosons decay leptonically. For these samples, Sherpa  calculates matrix elements at NLO for zero additional partons, at LO for one to three additional partons (with the exception of *ZZ* production, for which the one additional parton is also at NLO), and using PS for all parton multiplicities of four or more. All samples are normalised using the cross-section computed by the event generator.

Events with $t\bar{t}$ production in association with a vector boson are simulated using MG5_aMC@NLO $+$ Pythia 8 [50], using the NNPDF2.3 PDF set and the A14 tune, as described in Ref. [51].

Background contributions containing one prompt lepton and one misidentified (“fake”) lepton, arising from either a heavy-flavour hadron decay, photon conversion, jet misidentification or light-meson decay, are estimated using samples from MC simulation. The history of the stable particles in the generator-level record is used to identify fake leptons from these processes by identifying leptons that originated from hadrons. The majority ($\mathtt {\sim }$90%) of fake-lepton events originate from the single-lepton $t\bar{t}$ process, with smaller contributions arising from *W* + jets and $t\bar{t}$ + vector-boson events. *W* + jets events are simulated using Powheg-Box + Pythia 8 with the CT10 PDF set and the AZNLO tune [52]. The *t*-channel single-top quark process is generated using Powheg-Box v1 + Pythia 6 with the same parameters and PDF sets as those used for the nominal $t\bar{t}$ sample. EvtGen (v1.2.0) [53] is used for the heavy-flavour hadron decays in all samples. Other possible processes with fake leptons, such as multi-jet and Drell–Yan production, are negligible for the event selection used in this analysis.

## Object and event selection

This analysis utilises reconstructed electrons, muons, jets and missing transverse momentum (with magnitude $E_{\text {T}}^{\text {miss}}$). Electron candidates are identified by matching an inner-detector track to an isolated energy deposit in the electromagnetic calorimeter, within the fiducial region of transverse momentum $p_{\text {T}} > 25$ GeV and pseudorapidity $|\eta | < 2.47$. Electron candidates are excluded if the calorimeter cluster is within the transition region between the barrel and the endcap of the electromagnetic calorimeter, $1.37< |\eta | < 1.52$. Electrons are selected using a multivariate algorithm and are required to satisfy a likelihood-based quality criterion, in order to provide high efficiency and good rejection of fake electrons [54, 55]. Electron candidates must have tracks that pass the requirements of transverse impact parameter significance^2^ $|d_0^\text {sig}|<5$ and longitudinal impact parameter $|z_0 \sin \theta | < 0.5$ mm. Electrons must pass isolation requirements based on inner-detector tracks and topological clusters in the calorimeter which depend on $\eta$ and $p_{\text {T}}$. These requirements result in an isolation efficiency of 95% for an electron $p_{\text {T}}$ of 25 GeV and 99% for an electron $p_{\text {T}}$ above 60 GeV when determined in simulated $Z\rightarrow e^{+}e^{-}$ events. The fake-electron rate determined in simulated $t\bar{t}$ events is 2%. Electrons that share a track with a muon are discarded. Double counting of electron energy deposits as jets is prevented by removing the closest jet within $\Delta R = 0.2$ of a reconstructed electron. Following this, the electron is discarded if a jet exists within $\Delta R = 0.4$ of the electron to ensure sufficient separation from nearby jet activity.

Muon candidates are identified from muon-spectrometer tracks that match tracks in the inner detector, with $p_{\text {T}} > 25$ GeV and $|\eta | < 2.5$ [56]. The tracks of muon candidates are required to have a transverse impact parameter significance $|d_0^\text {sig}|<3$ and longitudinal impact parameter $|z_0 \sin \theta | < 0.5$ mm. Muons must satisfy quality criteria and isolation requirements based on inner-detector tracks and topological clusters in the calorimeter which depend on $\eta$ and $p_{\text {T}}$. These requirements reduce the contributions from fake muons and provide the same efficiency as for electrons when determined in simulated $t\bar{t}$ events. Muons may leave energy deposits in the calorimeter that could be misidentified as a jet, so jets with fewer than three associated tracks are removed if they are within $\Delta R = 0.4$ of a muon. Muons are discarded if they are separated from the nearest jet by $\Delta R < 0.4$ to reduce the background from muons from heavy-flavour hadron decays inside jets.

Jets are reconstructed with the anti-$k_t$ algorithm [57, 58], using a radius parameter of $R = 0.4$, from topological clusters of energy deposits in the calorimeters. Jets are accepted within the range $p_{\text {T}} > 25$ GeV and $|\eta | < 2.5$, and are calibrated using simulation with corrections derived from data [59]. Jets likely to originate from pile-up are suppressed using a multivariate jet-vertex-tagger (JVT) [60, 61] for candidates with $p_{\text {T}} < 60$ GeV and $|\eta | < 2.4$. Jets are identified as candidates for containing *b*-hadrons using a multivariate discriminant [62], which uses track impact parameters, track invariant mass, track multiplicity and secondary vertex information to discriminate *b*-jets from light-quark or gluon jets (light jets). The average *b*-tagging efficiency is 76%, with a purity of 90%, for *b*-jets in simulated dileptonic $t\bar{t}$ events.

$E_{\text {T}}^{\text {miss}}$ is reconstructed using calibrated electrons, muons and jets [63], where the electrons and muons are required to satisfy the selection criteria above. Tracks associated with the primary vertex are used for the computation of $E_{\text {T}}^{\text {miss}}$ from energy not associated with electrons, muons or jets. The primary vertex is defined as the vertex with the highest sum of $p_{\text {T}}^2$ of tracks associated with it.

Signal events are selected by requiring exactly one electron and one muon of opposite electric charge, and at least two jets, at least one of which must be *b*-tagged. No requirements are made on the $E_{\text {T}}^{\text {miss}}$ in the event. Using this selection, 85% of events are expected to be $t\bar{t}$ events. The other processes that pass the signal selection are Drell–Yan ($Z/\gamma ^{*}\rightarrow \tau ^{+}\tau ^{-}$), diboson and single-top quark (*Wt*) production and fake-lepton events.

**Fig. 1:**
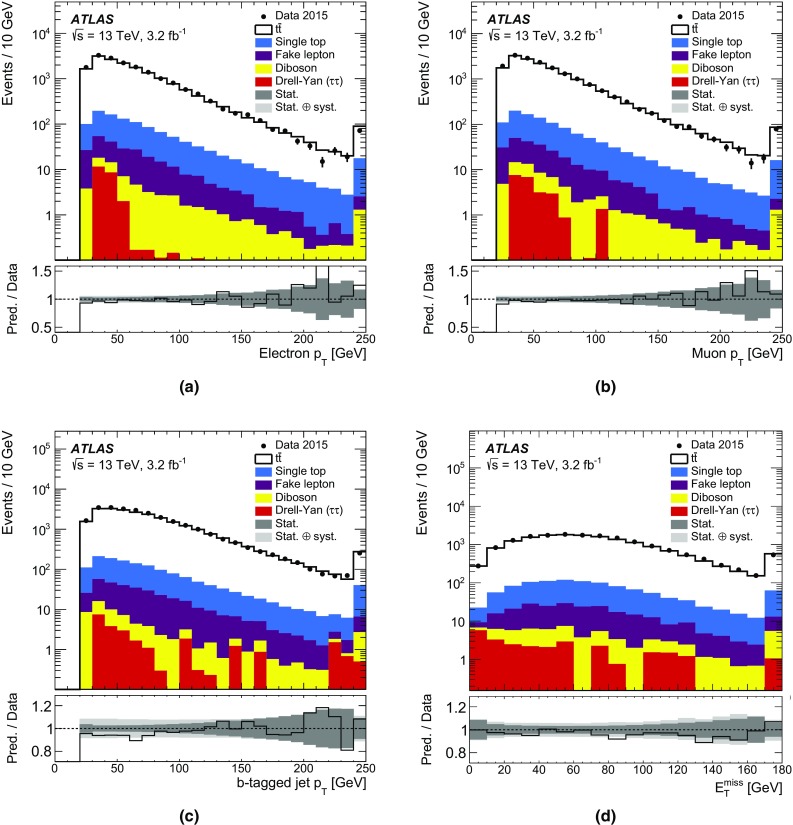
Kinematic distributions for the electron $p_{\text {T}}$ (**a**), muon $p_{\text {T}}$ (**b**), *b*-jet $p_{\text {T}}$ (**c**), and $E_{\text {T}}^{\text {miss}}$ (**d**) for the $e^{{\pm }}\mu ^{\mp }$ signal selection. In all figures, the rightmost bin also contains events that are above the *x*-axis range. The *dark uncertainty bands* in the ratio plots represent the statistical uncertainties while the *light uncertainty bands* represent the statistical, systematic and luminosity uncertainties added in quadrature. The uncertainties quoted include uncertainties from leptons, jets, missing transverse momentum, background modelling and pile-up modelling. They do not include uncertainties from PDF or signal $t\bar{t}$ modelling

The event yields after the signal selection are listed in Table 1. The number of events observed in the signal region exceeds the prediction, but the excess is within the uncertainties. Distributions of lepton and jet $p_{\text {T}}$ and $E_{\text {T}}^{\text {miss}}$ are shown in Fig. 1. The $t\bar{t}$ contribution is normalised using the predicted cross-section, calculated with the Top++2.0 program at next-to-next-to-leading order in perturbative QCD, including soft-gluon resummation to next-to-next-to-leading-logarithm order [6] and assuming a top-quark mass of 172.5 GeV. The data and prediction agree within the total uncertainty for all distributions. The $p_{\text {T}}$ observables show a small deficit in the simulation prediction at low $p_{\text {T}}$ which was found to be correlated with the modelling of the top-quark $p_{\text {T}}$.

**Table 1:** Event yields in the signal selection, and after requiring that neutrino weighting (NW) reconstructs the event. The quoted uncertainties include uncertainties from leptons, jets, missing transverse momentum, luminosity, statistics, background modelling and pile-up modelling. They do not include uncertainties from PDF or signal $t\bar{t}$ modelling. The results and uncertainties are rounded according to recommendations from the Particle Data Group (PDG)

Process	Signal region	Signal region $+$ NW
$Z/\gamma ^{*}\rightarrow \tau ^{+}\tau ^{-}$	22 ${\pm }$ 9	10 ${\pm }$ 8
Diboson	44 ${\pm }$ 4	17 ${\pm }$ 2
Fake lepton	200 ${\pm }$ 60	150 ${\pm }$ 50
*Wt*	860 ${\pm }$ 60	480 ${\pm }$ 40
$t\bar{t}$	15,800 ${\pm }$ 900	13,300 ${\pm }$ 800
Expected	17,000 ${\pm }$ 900	13,900 ${\pm }$ 800
Observed	17,501	14,387

Particle-level objects are constructed using generator-level information in the MC simulation, using a procedure intended to correspond as closely as possible to the reconstructed object and event selection. Only objects in the MC simulation with a lifetime longer than $3 \times 10^{-11}$ s (stable) in the generator-level information are used. Particle-level electrons and muons are identified as those originating from a *W*-boson decay, including those via intermediate $\tau$ leptons. The four-momenta of each electron or muon is summed with the four-momenta of all radiated photons, excluding those from hadron decays, within a cone of size $\Delta R = 0.1$, and the resulting objects are required to have $p_{\text {T}} > 25$ GeV and $|\eta | < 2.5$. Particle-level jets are constructed using stable particles, with the exception of selected particle-level electrons and muons and particle-level neutrinos originating from *W*-boson decays, using the anti-$k_t$ algorithm with a radius parameter of $R = 0.4$, in the region $p_{\text {T}} > 25$ GeV and $|\eta | < 2.5$. Intermediate *b*-hadrons in the MC decay chain history are clustered in the stable-particle jets with their energies set to zero. If, after clustering, a particle-level jet contains one or more of these “ghost” *b*-hadrons, the jet is said to have originated from a *b*-quark. This technique is referred to as “ghost matching” [64]. Particle-level $E_{\text {T}}^{\text {miss}}$ is calculated using the vector transverse-momentum sum of all neutrinos in the event, excluding those originating from hadron decays, either directly or via a $\tau$ lepton.

Events are selected at the particle level in a fiducial phase space region with similar requirements to the phase space region at reconstruction level. Events are selected by requiring exactly one particle-level electron and one particle-level muon of opposite electric charge, and at least two particle-level jets, at least one of which must originate from a *b*-quark.

## Reconstruction

The *t*, $\bar{t}$, and $t\bar{t}$ are reconstructed using both the particle-level objects and the reconstructed objects in order to measure their kinematic distributions. The reconstructed system is built using the neutrino weighting (NW) method [65].

Whereas the individual four-momenta of the two neutrinos in the final state are not directly measured in the detector, the sum of their transverse momenta is measured as $E_{\text {T}}^{\text {miss}}$. The absence of the measured four-momenta of the two neutrinos leads to an under-constrained system that cannot be solved analytically. However, if additional constraints are placed on the mass of the top-quark, the mass of the *W* boson, and on the pseudorapidities of the two neutrinos, the system can be solved using the following equations:

1 $$\begin{aligned} (\ell _{1,2} + \nu _{1,2})^2= & {} m^2_W = (80.2\,\text {{GeV}})^2, \nonumber \\ (\ell _{1,2} + \nu _{1,2} + b_{1,2})^2= & {} m_t^2 = (172.5\,\text {{GeV}})^2,\nonumber \\ \eta (\nu ),~\eta (\bar{\nu })= & {} \eta _{1},~\eta _{2}, \end{aligned}$$

where $\ell _{1,2}$ are the charged leptons, $\nu _{1,2}$ are the neutrinos, and $b_{1,2}$ are the *b*-jets (or jets), representing four-momentum vectors, and $\eta _{1},~\eta _{2}$ are the assumed $\eta$ values of the two neutrinos. Since the neutrino $\eta$’s are unknown, many different assumptions of their values are tested. The possible values for $\eta (\nu )$ and $\eta (\bar{\nu })$ are scanned between $-5$ and 5 in steps of 0.2.

With the assumptions about $m_t$, $m_W$, and values for $\eta (\nu )$ and $\eta (\bar{\nu })$, Eq. (1) can now be solved, leading to two possible solutions for each assumption of $\eta (\nu )$ and $\eta (\bar{\nu })$. Only real solutions without an imaginary component are considered. The observed $E_{\text {T}}^{\text {miss}}$ value in each event is used to determine which solutions are more likely to be correct. A “reconstructed” $E_{\text {T}}^{\text {miss}}$ value resulting from the neutrinos for each solution is compared to the $E_{\text {T}}^{\text {miss}}$ observed in the event. If this reconstructed $E_{\text {T}}^{\text {miss}}$ value matches the observed $E_{\text {T}}^{\text {miss}}$ value in the event, then the solution with those values for $\eta (\nu )$ and $\eta (\bar{\nu })$ is likely to be the correct one. A weight is introduced in order to quantify this agreement:

2 $$\begin{aligned} w = \exp \left( \frac{-\Delta E^2_x}{2\sigma _x^2}\right) \cdot \exp \left( \frac{-\Delta E^2_y}{2\sigma ^2_y} \right) , \end{aligned}$$

where $\Delta E_{x,y}$ is the difference between the missing transverse momentum computed from Eq. (1) and the observed missing transverse momentum in the *x*–*y* plane and $\sigma _{x,y}$ is the resolution of the observed $E_{\text {T}}^{\text {miss}}$ in the detector in the *x*–*y* plane. The assumption for $\eta (\nu )$ and $\eta (\bar{\nu })$ that gives the highest weight is used to reconstruct the *t* and $\bar{t}$ for that event. The $E_{\text {T}}^{\text {miss}}$ resolution is taken to be 15 GeV for both the *x* and *y* directions [63]. This choice has little effect on which solution is picked in each event. The highest-weight solution remains the same regardless of the choice of $\sigma _{x,y}$.

In each event, there may be more than two jets and therefore many possible combinations of jets to use in the kinematic reconstruction. In addition, there is an ambiguity in assigning a jet to the *t* or to the $\bar{t}$ candidate. In events with only one *b*-tagged jet, the *b*-tagged jet and the highest-$p_{\text {T}}$ non-*b*-tagged jet are used to reconstruct the *t* and $\bar{t}$, whereas in events with two or more *b*-tagged jets, the two *b*-tagged jets with the highest weight from the *b*-tagging algorithm are used.

Equation (1) cannot always be solved for a particular assumption of $\eta (\nu )$ and $\eta (\bar{\nu })$. This can be caused by misassignment of the input objects or through mismeasurement of the input object four-momenta. It is also possible that the assumed $m_t$ is sufficiently different from the true value to prevent a valid solution for that event. To mitigate these effects, the assumed value of $m_t$ is varied between the values of 168 and 178 GeV, in steps of 1 GeV, and the $p_{\text {T}}$ of the measured jets are smeared using a Gaussian function with a width of 10% of their measured $p_{\text {T}}$. This smearing is repeated 20 times. This allows the NW algorithm to shift the four-momenta (of the electron, muon and the two jets) and $m_t$ assumption to see if a solution can be found. The solution which produces the highest *w* is taken as the reconstructed system.

**Fig. 2:**
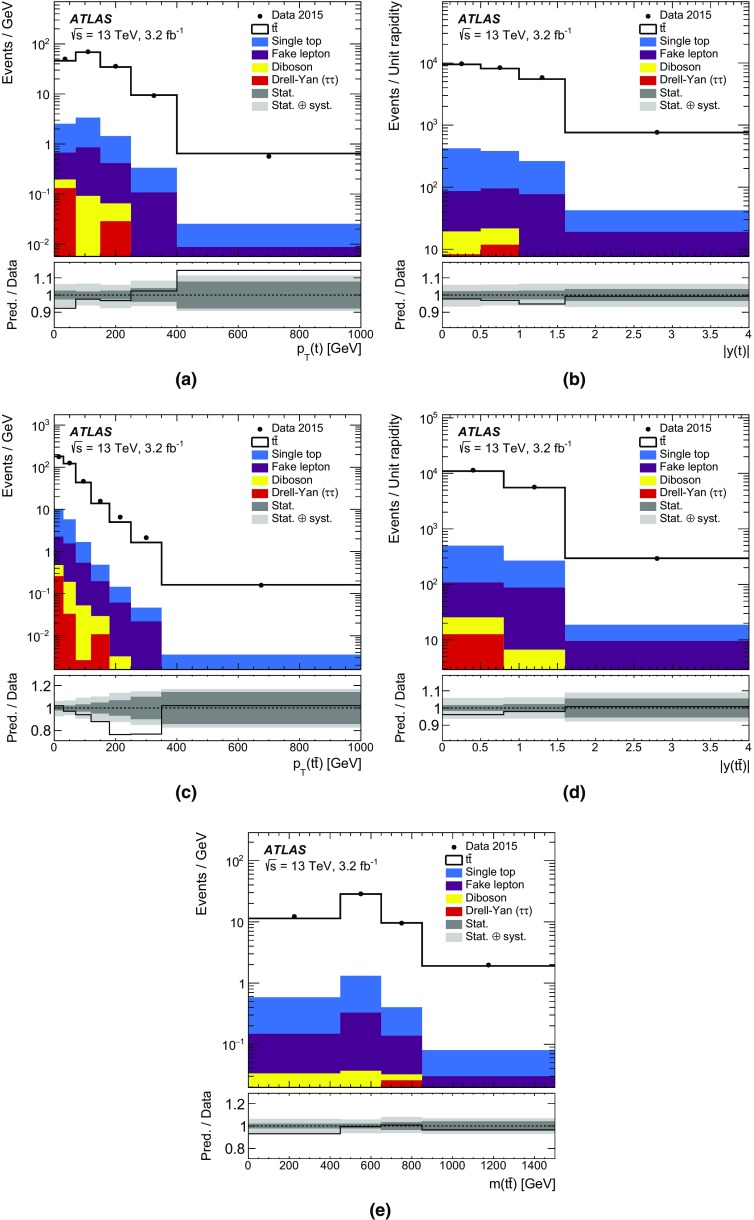
Kinematic distributions for the $p_{\text {T}} (t)$ (**a**), |*y*(*t*)| (**b**), $p_{\text {T}} (t\bar{t})$ (**c**), $t\bar{t}$ $|y_{t\bar{t}}|$ (**d**), and $m(t\bar{t})$ (**e**) after reconstruction of the $t\bar{t}$ system. In all figures, the rightmost bin also contains events that are above the *x*-axis range. The *uncertainty bands* represent the statistical uncertainties (*dark*) and the statistical, systematic and luminosity uncertainties added in quadrature (*light*). The uncertainties quoted include uncertainties on leptons, jets, $E_{\text {T}}^{\text {miss}}$, background and pile-up modelling, and luminosity. They do not include uncertainties on PDF or signal $t\bar{t}$ modelling

For a fraction of events, even smearing does not help to find a solution. Such events are not included in the signal selection and are counted as an inefficiency of the reconstruction. For the signal $t\bar{t}$ MC samples, the inefficiency is $\mathtt {\sim }$20%. Due to the implicit assumptions about the $m_t$ and $m_W$, the reconstruction inefficiency found in simulated background samples is much higher ($\mathtt {\sim }$40% for *Wt* and Drell–Yan processes) and leads to a suppression of background events. Table 1 shows the event yields before and after reconstruction in the signal region. The purity of $t\bar{t}$ events increases after reconstruction. The distributions of the experimental observables after reconstruction are shown in Fig. 2.

Particle-level *t*, $\bar{t}$, and $t\bar{t}$ objects are reconstructed following the prescriptions from the LHCTopWG, with the exception that only events with at least one *b*-tagged jet are allowed. Events are required to have exactly two leptons of opposite-sign electric charge (one electron and one muon), and at least two jets. The *t* and $\bar{t}$ are reconstructed by considering the two particle-level neutrinos with the highest $p_{\text {T}}$ and the two particle-level charged leptons. The charged leptons and the neutrinos are paired such that $|m_{\nu _1,\ell _1} - m_W| + |m_{\nu _2,\ell _2} - m_W|$ is minimised. These pairs are then used as pseudo *W* bosons and are paired with particle-level jets such that $|m_{W_1,j_1} - m_t| + |m_{W_2,j_2} - m_t|$ is minimised, where at least one of the jets must be *b*-tagged. In cases where only one particle-level *b*-jet is present, the particle-level jet with the highest $p_{\text {T}}$ among the non-*b*-tagged jets is used as the second jet. In cases with two particle-level *b*-jets, both are taken. In the rare case of events with more than two particle-level *b*-jets, the two highest-$p_{\text {T}}$ particle-level *b*-jets are used. The particle-level $t\bar{t}$ object is constructed using the sum of the four-momenta of the particle-level *t* and $\bar{t}$.

## Unfolding

To obtain the absolute and normalised differential cross-sections in the fiducial phase space region (see Sect. 4) with respect to the $t\bar{t}$ system variables, the distributions are unfolded to particle level using an iterative Bayesian method [66] implemented in the RooUnfold package [67]. In the unfolding, background-subtracted data are corrected for detector acceptance and resolution effects as well as for the efficiency to pass the event selection requirements in order to obtain the absolute differential cross-sections. The fiducial differential cross-sections are divided by the measured total cross-section, obtained by integrating over all bins in the differential distribution, in order to obtain the normalised differential cross-sections.

The differential cross-sections are calculated using the equation:

3 $$\begin{aligned} \frac{\text {d}\sigma _{t\bar{t}}}{\text {d}X_i} = \frac{1}{\mathcal {L} \cdot \mathcal {B} \cdot \Delta X_i \cdot \epsilon _i} \cdot \sum _{j} R^{-1}_{ij} \cdot \epsilon ^{\text {fid}}_j \cdot (N_j^{\text {obs}}-N_j^{\text {bkg}}) \text {,} \end{aligned}$$

where *i* indicates the bin for the observable *X*, $\Delta X_i$ is the width of bin *i*, $\mathcal {L}$ is the integrated luminosity, $\mathcal {B}$ is the branching ratio of the process ($t\bar{t} \rightarrow b \bar{b} e^{{\pm }} \nu _e \mu ^{\mp } \nu _{\mu }$), *R* is the response matrix, $N_j^\text {obs}$ is the number of observed events in data in bin *j*, and $N_j^\text {bkg}$ is the estimated number of background events in bin *j*. The efficiency parameter, $\epsilon _i$ ($\epsilon_j^{\text{fid}}$), is used to correct for events passing the reconstructed (fiducial) event selection but not the fiducial (reconstructed) selection.

**Fig. 3:**
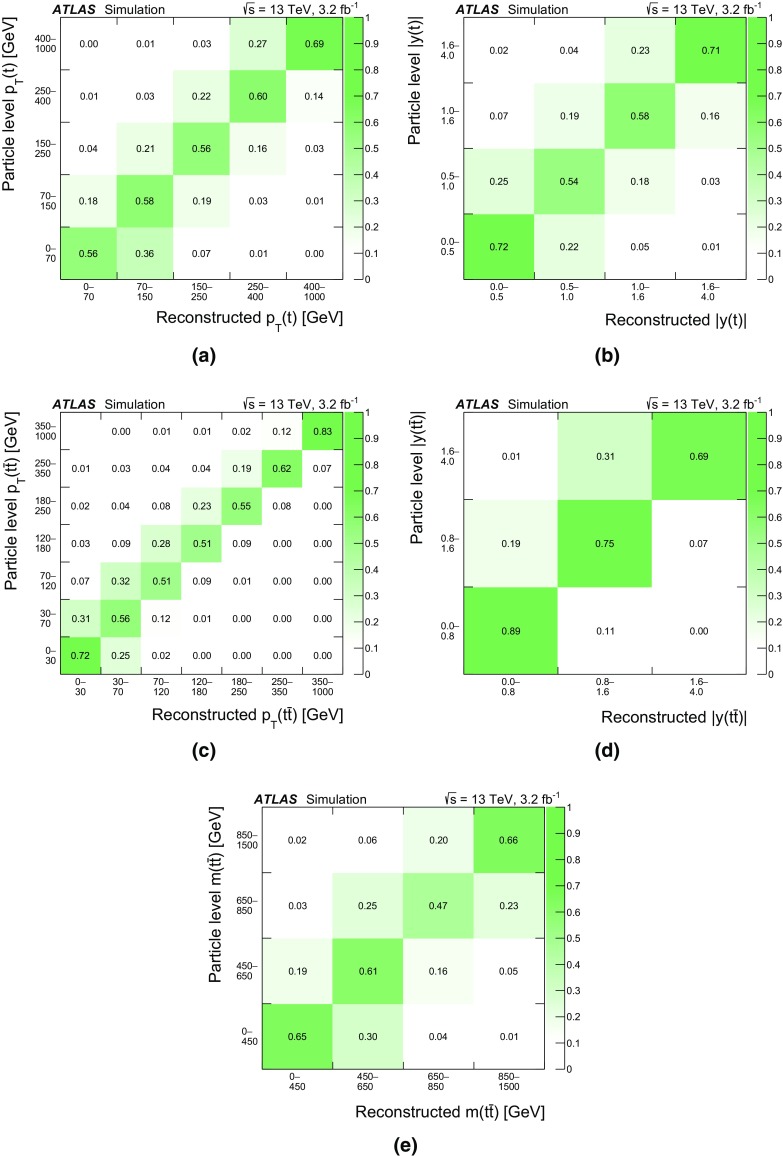
The response matrices for the observables obtained from the nominal $t\bar{t}$ MC, normalised by row to unity. *Each bin* shows the probability for a particle-level event in bin *j* to be observed in a reconstruction-level bin *i*. White corresponds to 0 probability and the *darkest green* to a probability of one, where the other probabilities lie in between those *shades*

The response matrix, *R*, describes the detector response, and is determined by mapping the bin-to-bin migration of events from particle level to reconstruction level in the nominal $t\bar{t}$ MC simulation. Figure 3 shows the response matrices that are used for each experimental observable, normalised such that the sum of entries in each row is equal to one. The values represent the fraction of events at particle level in bin *i* that are reconstructed in bin *j* at reconstruction level.

The binning for the observables is chosen such that approximately half of the events are reconstructed in the same bin at reconstruction level as at the particle level (corresponding to a value of approximately 0.5 in the diagonal elements of the migration matrix). Pseudo-data are constructed by randomly sampling events from the nominal $t\bar{t}$ MC sample, to provide a number of events similar to the number expected from data. These pseudo-data are used to establish the stability of unfolding with respect to the choice of binning with pull tests. The binning choice must result in pulls consistent with a mean of zero and a standard deviation of one, within uncertainties. The choice of binning does not introduce any bias or underestimation of the statistical uncertainties. The number of iterations used in the iterative Bayesian unfolding is also optimised using pseudo-experiments. Iterations are performed until the $\chi ^2$ per degree of freedom, calculated by comparing the unfolded pseudo-data to the corresponding generator-level distribution for that pseudo-data set, is less than unity. The optimum number of iterations is determined to be six. Tests are performed to establish that the unfolding procedure is able to successfully unfold distributions other than those predicted by the nominal MC simulation.

## Systematic uncertainties

**Fig. 4:**
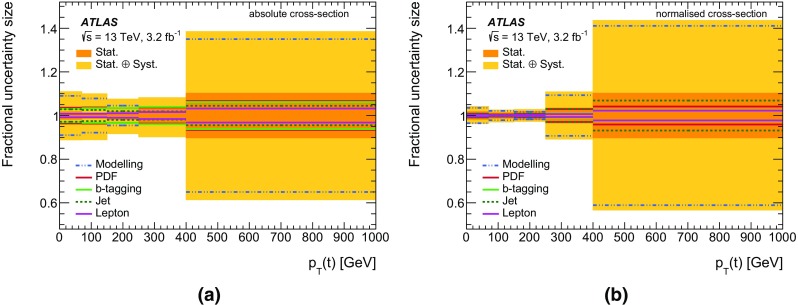
Summary of the fractional size of the absolute (**a**) and normalised (**b**) fiducial differential cross-sections as a function of $p_{\text {T}}(t)$. Systematic uncertainties which are symmetric are represented by *solid lines* and asymmetric uncertainties are represented by *dashed* or *dot*–*dashed lines*. Systematic uncertainties from common sources, such as modelling of the $t\bar{t}$ production, have been grouped together. Uncertainties due to luminosity or background modelling are not included. The statistical and total uncertainty sizes are indicated by the *shaded bands*

The measured differential cross-sections are affected by systematic uncertainties arising from detector response, signal modelling, and background modelling. The contributions from various sources of uncertainty are described in this section. Summaries of the sources of uncertainty for the absolute and normalised differential cross-sections for the $p_{\text {T}}(t)$ are presented in Tables 2 and 3. The total systematic uncertainties are calculated by summing all of the individual systematic uncertainties in quadrature and the total uncertainty is calculated by summing the systematic and statistical uncertainties in quadrature. The effect of different groups of systematic uncertainties is shown graphically for $p_{\text {T}}(t)$ in Fig. 4.

### Signal modelling uncertainties

The following systematic uncertainties related to the modelling of the $t\bar{t}$ system in the MC generators are considered: the choice of matrix-element generator, the hadronisation model, the choice of PDF, and the amount of initial- and final-state radiation.

Each source is estimated by using a different MC sample in the unfolding procedure. In particular, a chosen baseline MC sample is unfolded using response matrices and corrections derived from an alternative sample. The difference between the unfolded distribution in the baseline sample and the true distribution in the baseline sample is taken as the systematic uncertainty due to the signal modelling.

The choice of NLO generator (MC generator) affects the kinematic properties of the simulated $t\bar{t}$ events and the reconstruction efficiencies. To estimate this uncertainty, a comparison between Powheg-Box and MG5_aMC@NLO (both using Herwig++ for the parton-shower simulation) is performed, with the Powheg-Box sample used as the baseline. The resulting systematic shift is used to define a symmetric uncertainty, where deviations from the nominal sample are also considered to be mirrored in the opposite direction, resulting in equal and opposite symmetric uncertainties (called symmetrising).

To evaluate the uncertainty arising from the choice of parton-shower algorithm, a sample generated using Powheg-Box + Pythia 6 is compared to the alternative sample generated with Powheg-Box + Herwig++, where both samples use “fast simulation”. The resulting uncertainty is symmetrised. The choices of NLO generator and parton-shower algorithm are dominant sources of systematic uncertainty in all observables.

The uncertainty due to the choice of PDF is evaluated using the PDF4LHC15 prescription [68]. The prescription utilises 100 eigenvector shifts derived from fits to the CT14 [69], MMHT [69] and NNPDF3.0 [70] PDF sets (PDF4LHC 100). The nominal MC sample used in the analysis is generated using the CT10 PDF set. Therefore, the uncertainty is taken to be the standard deviation of all eigenvector variations summed in quadrature with the difference between the central values of the CT14 and CT10 PDF sets (PDF extrapolation). The resulting uncertainty is symmetrised. Both PDF-based uncertainties contribute as one of the dominant systematic uncertainties.

Uncertainties arising from varying the amount of initial- and final-state radiation (radiation scale), which alters the jet multiplicity in events and the transverse momentum of the $t\bar{t}$ system, are estimated by comparing the nominal Powheg-Box + Pythia 6 sample to samples generated with high and low radiation settings, as discussed in Sect. 3. The uncertainty is taken as the difference between the nominal and the increased radiation sample, and the nominal and the decreased radiation sample. The initial- and final-state radiation is a significant source of uncertainty in the absolute cross-section measurements but only a moderate source of uncertainty in the normalised cross-sections.

### Background modelling uncertainties

The uncertainties in the background processes are assessed by repeating the full analysis using pseudo-data sets and by varying the background predictions by one standard deviation of their nominal values. The difference between the nominal pseudo-data set result and the shifted result is taken as the systematic uncertainty.

Each background prediction has an uncertainty associated with its theoretical cross-section. The cross-section for the *Wt* process is varied by ${\pm }$5.3% [42], the diboson cross-section is varied by ${\pm }$6%, and the Drell–Yan $Z/\gamma ^*\rightarrow \tau ^+\tau ^-$ background is varied by ${\pm }$5% based on studies of different MC generators. A 30% uncertainty is assigned to the normalisation of the fake-lepton background based on comparisons between data and MC simulation in a fake-dominated control region, which is selected in the same way as the $t\bar{t}$ signal region but the leptons are required to have same-sign electric charges.

An additional uncertainty is evaluated for the *Wt* process by replacing the nominal DR sample with a DS sample, as discussed in Sect. 3, and taking the difference between the two as the systematic uncertainty.

### Detector modelling uncertainties

Systematic uncertainties due to the modelling of the detector response affect the signal reconstruction efficiency, the unfolding procedure, and the background estimation. In order to evaluate their impact, the full analysis is repeated with variations of the detector modelling and the difference between the nominal and the shifted results is taken as the systematic uncertainty.

The uncertainties due to lepton isolation, trigger, identification, and reconstruction requirements are evaluated in 2015 data using a tag-and-probe method in leptonically decaying *Z*-boson events [56]. These uncertainties are summarised as “Lepton” in Tables 2 and 3.

The uncertainties due to the jet energy scale and resolution are extrapolated to $\sqrt{s} = 13$ TeV using a combination of test beam data, simulation and $\sqrt{s} = 8$ TeV dijet data [59]. To account for potential mismodelling of the JVT distribution in simulation, a 2% systematic uncertainty is applied to the jet efficiency. These uncertainties are summarised as “Jet” in Tables 2 and 3. Uncertainties due to *b*-tagging, summarised under “*b*-tagging”, are determined using $\sqrt{s}=8$ TeV data as described in Ref. [71] for *b*-jets and Ref. [72] for *c*- and light-jets, with additional uncertainties to account for the presence of the new Insertable B-Layer detector and the extrapolation from $\sqrt{s}=8$ TeV to $\sqrt{s}=13$ TeV [62].

The systematic uncertainty due to the track-based terms (i.e. those tracks not associated with other reconstructed objects such as leptons and jets) used in the calculation of $E_{\text {T}}^{\text {miss}}$ is evaluated by comparing the $E_{\text {T}}^{\text {miss}}$ in $Z\rightarrow \mu \mu$ events, which do not contain prompt neutrinos from the hard process, using different generators. Uncertainties associated with energy scales and resolutions of leptons and jets are propagated to the $E_{\text {T}}^{\text {miss}}$ calculation.

The uncertainty due to the integrated luminosity is ${\pm } 2.1$%. It is derived, following a methodology similar to that detailed in Ref. [73], from a calibration of the luminosity scale using *x*–*y* beam-separation scans performed in August 2015. The uncertainty in the pile-up reweighting is evaluated by varying the scale factors by ${\pm }1\sigma$ based on the reweighting of the average number of interactions per bunch crossing.

The uncertainties due to lepton and $E_{\text {T}}^{\text {miss}}$ modelling are not large for any observable. For the absolute cross-sections, the uncertainty due to luminosity is not a dominant systematic uncertainty, and this uncertainty mainly cancels in the normalised cross-sections. The luminosity uncertainty does not cancel fully since it affects the background subtraction. The uncertainty due to jet energy scale and JVT is a significant source of uncertainty in the absolute cross-sections and in some of the normalised cross-sections such as for $p_{\text {T}}(t\bar{t})$. The uncertainties due to the limited number of MC events are evaluated using pseudo-experiments. The data statistical uncertainty is evaluated using the full covariance matrix from the unfolding.

**Table 2:** Summary of the sources of uncertainty in the absolute fiducial differential cross-section as a function of $p_{\text {T}}(t)$. The uncertainties are presented as a percentage of the measured cross-section in each bin. Entries with 0.0 are uncertainties that are less than 0.05 in magnitude. For systematic uncertainties that have only one variation, ${{\pm }}({\mp })$ indicate that the systematic shift is positive (negative) and then symmetrised. All uncertainties are rounded to two digits

$p_{\text {T}}(t)$	0–70 GeV	70–150 GeV	150–250 GeV	250–400 GeV	400–1000 GeV
Source	Systematic uncertainty (%)				
Radiation scale	$+$4.0 −3.9	$+$1.1 −3.9	$+$1.9 −3.5	$+$1.4 −5.0	$+$5.0 −5.4
MC generator	${\mp }$0.9	${\mp }$1.2	${\mp }$1.4	${\pm }$1.6	${\mp }$6.7
PDF extrapolation	${\mp }$2.9	${\mp }$2.8	${\mp }$1.9	${\mp }$0.3	${\mp }$2.4
PDF4LHC 100	${\pm }$2.2	${\pm }$2.5	${\pm }$2.8	${\pm }$3.7	${\pm }$6.1
Parton shower	${\mp }$8.0	${\mp }$7.7	${\mp }$3.9	${\pm }$3.1	${\pm }$34
Background	$+$0.3 −0.5	$+$0.2 −0.4	${\pm }$0.2	${\pm }$0.2	$+$0.4 −1.5
Pile-up	$+$0.7 −1.4	$+$0.2 −0.6	$+$0.0 −0.4	$+$0.0 −0.4	$+$4.1 −0.0
Lepton	$+$0.8 −0.7	${\pm }$0.8	${\pm }$1.0	${\pm }$1.6	$+$3.2 −3.0
*b*-tagging	$+$3.1 −3.6	$+$3.4 −3.9	$+$3.4 −4.0	$+$4.0 −4.7	$+$6.2 −7.2
Jet	${\pm }$2.8	$+$2.6 −3.4	$+$2.0 −1.8	$+$1.9 −1.1	$+$4.5 −5.1
$E^{\text {m}iss}_{\text {T}}$	$+$0.2 −0.1	${\pm }$0.1	$+$0.2 −0.1	$+$0.3 −0.5	$+$1.0 −0.3
Luminosity	$+$2.0 −2.1	$+$2.1 −2.2	$+$2.1 −2.2	$+$2.3 −2.4	$+$3.0 −3.1
MC stat. unc.	${\pm }$0.4	${\pm }$0.3	${\pm }$0.5	${\pm }$0.9	${\pm }$3.2
Total syst. unc.	$+$11 −11	$+$9 −11	$+$7.3 −8.1	$+$7.5 −9.1	$+$37 −37
Data statistics	${\pm }$1.8	${\pm }$1.3	${\pm }$1.8	${\pm }$3.4	${\pm }$10
Total uncertainty	$+$11 −11	$+$10 −11	$+$7.5 −8.3	$+$8.2 −9.8	$+$38 −39

**Table 3:** Summary of the sources of uncertainty in the normalised fiducial differential cross-section as a function of $p_{\mathrm{T}}(t)$. The uncertainties are presented as a percentage of the measured cross-section in each bin. Entries with 0.0 are uncertainties that are less than 0.05 in magnitude. For systematic uncertainties that have only one variation, ${{\pm }}({{\mp }})$ indicate that the systematic shift is positive (negative) and then symmetrised. All uncertainties are rounded to two digits

$p_{\mathrm{T}}(t)$	0–70 GeV	70–150 GeV	150–250 GeV	250–400 GeV	400–1000 GeV
Source	Systematic uncertainty (%)				
Radiation scale	$+$2.1 −0.3	$+$0.0 −1.1	$+$0.4 −0.3	$+$0.0 −1.2	$+$2.1 −0.0
MC generator	${\pm }$0.2	${\mp }$0.2	${\mp }$0.4	${\pm }$2.7	${\mp }$5.4
PDF extrapolation	${\mp }$0.5	${\mp }$0.4	${\pm }$0.4	${\pm }$2.4	${\pm }$0.8
PDF4LHC 100	${\pm }$0.6	${\pm }$0.3	${\pm }$0.5	${\pm }$1.7	${\pm }$4.0
Parton shower	${\mp }$2.8	${\mp }$2.1	${\pm }$1.6	${\pm }$8.9	${\pm }$41
Background	$+$0.1 −0.2	$+$0.0 −0.1	$+$0.3 −0.0	$+$0.3 −0.1	$+$0.1 −1.2
Pile-up	$+$0.4 −0.8	${\pm }$0.0	$+$0.3 −0.2	$+$0.8 −0.7	$+$5.1 −0.0
Lepton	$+$0.4 −0.3	$+$0.1 −0.3	$+$0.3 −0.1	${\pm }$0.7	$+$2.3 −1.9
*b*-tagging	${\pm }$0.2	${\pm }$0.2	${\pm }$0.2	${\pm }$0.9	$+$2.3 −2.4
Jet	$+$0.9 −0.8	$+$0.4 −1.0	$+$0.8 −0.6	$+$3.0 −2.4	$+$6.9 −7.3
$E^{\mathrm{m}iss}_{\mathrm{T}}$	$+$0.2 −0.1	$+$0.0 −0.1	$+$0.2 −0.1	$+$0.3 −0.5	$+$1.0 −0.4
Luminosity	${\pm }$0.0	${\pm }$0.0	${\pm }$0.0	${\pm }$0.0	${\pm }$0.0
MC stat. unc.	${\pm }$0.0	${\pm }$0.2	${\pm }$0.0	${\pm }$0.4	${\pm }$2.6
Total syst. unc.	$+$3.8 −3.2	$+$2.2 −2.7	$+$2.1 −2.0	$+$10 −10	$+$42 −42
Data statistics	${\pm }$1.8	${\pm }$1.3	${\pm }$1.8	${\pm }$3.4	${\pm }$10
Total uncertainty	$+$4.2 −3.6	$+$2.6 −2.9	$+$2.8 −2.7	$+$11 −11	$+$44 −43

## Results

The unfolded particle-level distributions for the absolute and normalised fiducial differential cross-sections are presented in Table 4. The total systematic uncertainties include all sources discussed in Sect. 7.

The unfolded normalised data are used to compare with different generator predictions. The significance of the differences of various generators, with respect to the data in each observable, are evaluated by calculating the $\chi ^2$ and determining *p*-values using the number of degrees of freedom (NDF). The $\chi ^2$ is determined using:

4 $$\begin{aligned} \chi ^2 = S^{\text {T}}_{(N - 1)} \cdot \text {Cov}^{-1}_{(N - 1)} \cdot S_{(N - 1)} \text {,} \end{aligned}$$

where $\text {Cov}^{-1}$ is the inverse of the full bin-to-bin covariance matrix, including all statistical and systematic uncertainties, *N* is the number of bins, and *S* is a column vector of the differences between the unfolded data and the prediction. The NDF is equal to the number of bins minus one in the observable for the normalised cross-sections. In $\text {Cov}$ and *S*, a single bin is removed from the calculation to account for the normalisation of the observable, signified by the $(N - 1)$ subscript. The $\chi ^2$, NDF, and associated *p*-values are presented in Table 5 for the normalised cross-sections. Most generators studied agree with the unfolded data in each observable within the experimental uncertainties, with the exception of the Powheg-Box + Herwig++ MC simulation, which differs significantly from the data in both $p_{\text {T}}(t)$ and $m(t\bar{t})$.

**Fig. 5:**
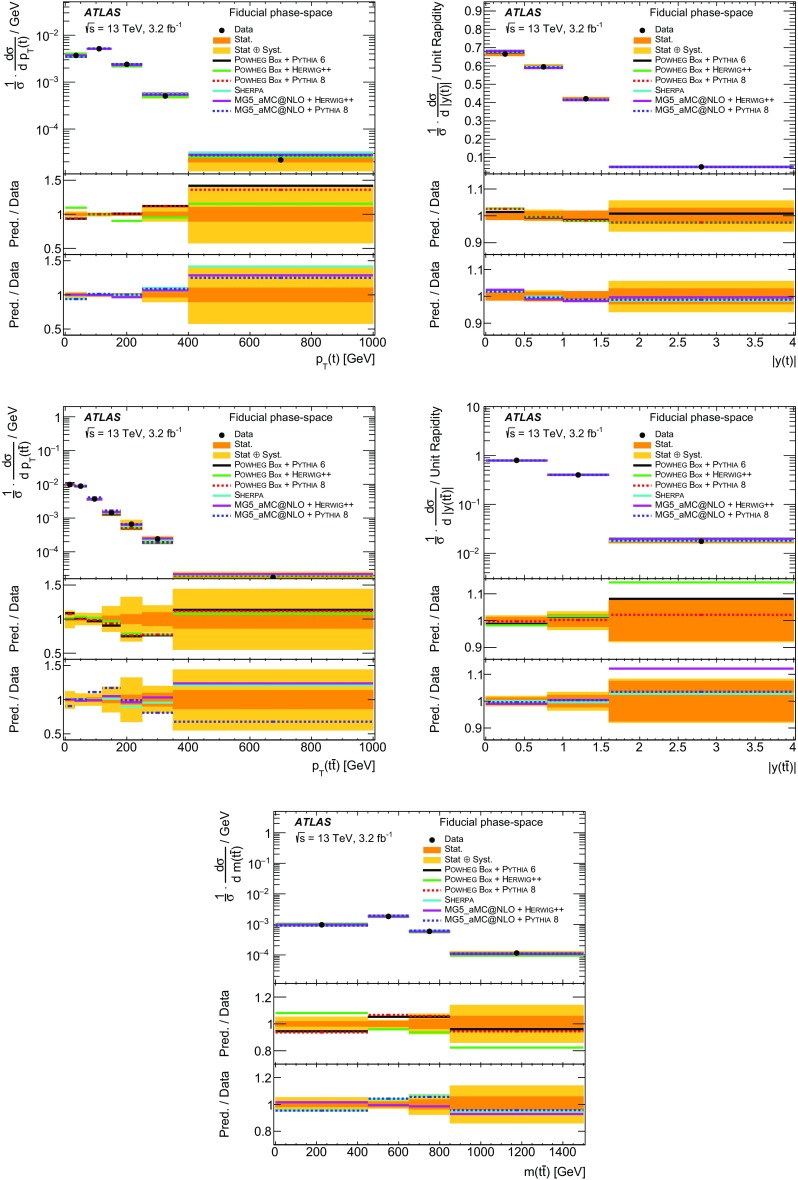
The measured normalised fiducial differential cross-sections compared to predictions from Powheg-Box (*top ratio panel*), MG5_aMC@NLO, and Sherpa (*bottom ratio panel*) interfaced to various parton shower programs

The normalised differential cross-sections for all observables are compared to predictions of different MC generators in Fig. 5.

The Powheg-Box generator tends to predict a harder $p_{\text {T}}(t)$ spectrum for the top quark than is observed in data, although the data are still consistent with the prediction within the experimental uncertainties. The MG5_aMC@NLO generator appears to agree better with the observed $p_{\text {T}}(t)$ spectrum, particularly when interfaced to Herwig++. For the $p_{\text {T}}(t\bar{t})$ spectrum, again little difference is observed between Powheg-Box + Pythia 6 and Pythia 8, and both generally predict a softer spectrum than the data but are also consistent within the experimental uncertainties. The MG5_aMC@NLO generator, interfaced to Pythia 8 or Herwig++ seems to agree with the data at low to medium values of $p_{\text {T}}$ but MG5_aMC@NLO + Herwig++ disagrees at higher values. For the $m(t\bar{t})$ observable, although the uncertainties are quite large, predictions from Powheg-Box interfaced to Pythia 6 or Pythia 8 and the MG5_aMC@NLO + Pythia 8 prediction seem higher than the observed data around 600 GeV. For the rapidity observables, all MC predictions appear to agree with the observed data, except for the high $|y(t\bar{t})|$ region, where some of the predictions are slightly higher than the data.

**Table 4:** Summary of the measured absolute ($\frac{\text {d}\sigma _{t\bar{t}}}{\text {d}X}$) and normalised ($\frac{1}{\sigma _{t\bar{t}}}\frac{\text {d}\sigma _{t\bar{t}}}{\text {d}X}$) differential cross-sections, along with the relative statistical (Stat.) and systematic (Syst.) uncertainties for both the absolute (abs.) and normalised (norm.) cross-sections. The results and uncertainties are rounded according to recommendations from the Particle Data Group (PDG)

*X*	$\frac{\text {d}\sigma _{t\bar{t}}}{\text {d}X}$ $[\frac{\text {pb}}{\text {GeV}}]$	$\frac{1}{\sigma _{t\bar{t}}}\frac{\text {d}\sigma _{t\bar{t}}}{\text {d}X}$ $[\frac{1}{\text {GeV}}]$	Stat. (abs.) (%)	Stat. (norm.) (%)	Syst. (abs.) (%)	Syst. (norm.) (%)
$$p_{\text {T}}(t)$$ (GeV)						
0–70	7.1	0.371	${\pm }$1.8	${\pm }$1.7	$+$11 −11	$+$4 −3.2
70–150	9.9	0.515	${\pm }$1.3	${\pm }$1.2	$+$10 −11	$+$2.3 −2.7
150–250	4.61	0.239	${\pm }$1.8	${\pm }$1.7	$+$7 −8	$+$2.1 −2.0
250–400	0.97	0.051	${\pm }$3.4	${\pm }$3.3	$+$7 −9	$+$10 −11
400–1000	0.042	0.0022	${\pm }$10	${\pm }$9	$+$40 −40	$+$40 −40
$$p_{\text {T}}(t\bar{t})$$ (GeV)						
0–30	9.6	0.99	${\pm }$2.2	${\pm }$2.0	$+$15 −16	$+$12 −13
30–70	8.6	0.88	${\pm }$1.9	${\pm }$1.7	$+$8 −8	$+$9 −9
70–120	3.6	0.368	${\pm }$3.0	${\pm }$2.7	$+$10 −11	$+$8 −9
120–180	0.139	0.143	${\pm }$5	${\pm }$5	$+$24 −24	$+$19 −18
180–250	0.064	0.066	${\pm }$7	${\pm }$6	$+$40 −40	$+$32 −32
250–350	0.023	0.024	${\pm }$10	${\pm }$9	$+$24 −24	$+$30 −19
350–1000	0.0017	0.0018	${\pm }$14	${\pm }$13	$+$50 −50	$+$40 −40
$$m(t\bar{t})$$ (GeV)						
0–450	0.94	0.097	${\pm }$1.8	${\pm }$1.6	$+$12 −13	$+$5 −5
450–650	1.76	0.183	${\pm }$2.0	${\pm }$1.9	$+$8 −9	$+$2.8 −3.0
650–850	0.57	0.059	${\pm }$4	${\pm }$3.3	$+$10 −12	$+$8 −8
850–1500	0.111	0.0115	${\pm }$6	${\pm }$5	$+$11 −11	$+$14 −14

*X*	$\frac{\text {d}\sigma _{t\bar{t}}}{\text {d}X}$ [pb]	$\frac{1}{\sigma _{t\bar{t}}}\frac{\text {d}\sigma _{t\bar{t}}}{\text {d}X}$	Stat. (abs.) (%)	Stat. (norm.) (%)	Syst. (abs.) (%)	Syst. (norm.) (%)
$|y(t\bar{t}$)|						
0.0–0.8	7.7	0.797	${\pm }$1.3	${\pm }$1.1	$+$8 −9	$+$1.8 −1.8
0.8–1.6	3.9	0.400	${\pm }$2.2	${\pm }$2.0	$+$9 −10	$+$3.4 −3.4
1.6–4.0	0.170	0.0176	${\pm }$7	${\pm }$7	$+$13 −13	$+$8 −8
|*y*(*t*)|						
0.0–0.5	12.9	0.665	${\pm }$1.5	${\pm }$1.4	$+$8 −10	$+$1.0 −1.3
0.5–1.0	11.5	0.595	${\pm }$1.6	${\pm }$1.5	$+$10 −10	$+$2.2 −1.9
1.0–1.6	8.1	0.421	${\pm }$1.8	${\pm }$1.7	$+$8 −9	$+$1.4 −1.2
1.6–4.0	0.95	0.0489	${\pm }$2.9	${\pm }$2.7	$+$8 −9	$+$6 −6

**Table 5:** $\chi ^2$ values between the normalised unfolded fiducial cross-section and various predictions from the MC simulation. The number of degrees of freedom (NDF) is equal to one less than the number of bins in the distribution. Powheg refers to Powheg-Box v2

Predictions	$p_{\text {T}}(t)$		|*y*(*t*)|		$p_{\text {T}}(t\bar{t})$		$|y(t\bar{t})|$		$m(t\bar{t})$	
	$\chi ^2$/NDF	*p*-value	$\chi ^2$/NDF	*p*-value	$\chi ^2$/NDF	*p*-value	$\chi ^2$/NDF	*p*-value	$\chi ^2$/NDF	*p*-value
Powheg $+$ Pythia 6	5.2/4	0.27	0.5/3	0.92	5.5/6	0.48	0.6/2	0.74	3.9/4	0.42
Powheg $+$ Pythia 8	4.6/4	0.33	1.3/3	0.73	5.1/6	0.53	0.0/2	1.00	5.7/4	0.22
Powheg $+$ Herwig++	14.6/4	0.01	1.4/3	0.71	4.1/6	0.66	1.0/2	0.61	12.0/4	0.02
MG5_aMC@NLO $+$ Herwig++	2.0/4	0.74	1.3/3	0.73	0.6/6	1.00	0.2/2	0.90	0.9/4	0.92
MG5_aMC@NLO $+$ Pythia 8	3.6/4	0.46	0.6/3	0.90	10.7/6	0.10	0.1/2	0.95	2.7/4	0.61
Sherpa	3.8/4	0.43	0.8/3	0.85	0.7/6	0.99	0.0/2	1.00	2.3/4	0.68
Powheg $+$ Pythia 6 (radHi)	7.8/4	0.10	0.6/3	0.90	0.9/6	0.99	0.4/2	0.82	3.8/4	0.43
Powheg $+$ Pythia 6 (radLow)	5.5/4	0.24	0.8/3	0.85	9.6/6	0.14	0.8/2	0.67	4.5/4	0.34

## Conclusions

Absolute and normalised differential top-quark pair-production cross-sections in a fiducial phase-space region are measured using 3.2 fb$^{-1}$ of $\sqrt{s} = 13$ TeV proton–proton collisions recorded by the ATLAS detector at the LHC in 2015. The differential cross-sections are determined in the $e^{{\pm }}\mu ^{{\mp }}$ channel, for the transverse momentum and the absolute rapidity of the top quark, as well as the transverse momentum, the absolute rapidity, and the invariant mass of the top-quark pair. The measured differential cross-sections are compared to predictions of NLO generators matched to parton showers and the results are found to be consistent with all models within the experimental uncertainties, with the exception of Powheg -Box + Herwig ++, which deviates from the data in the $p_{\text {T}}(t)$ and $m(t\bar{t})$ observables.

## Footnotes

1 ATLAS uses a right-handed coordinate system with its origin at the nominal interaction point (IP) in the centre of the detector and the *z*-axis along the beam pipe. The *x*-axis points from the IP to the centre of the LHC ring, and the *y*-axis points upwards. Cylindrical coordinates $(r, \phi )$ are used in the transverse plane, $\phi$ being the azimuthal angle around the *z*-axis. The pseudorapidity is defined in terms of the polar angle $\theta$ as $\eta = -\ln \tan (\theta / 2)$. Angular distance is measured in units of $\Delta R \equiv \sqrt{(\Delta \eta )^{2} + (\Delta \phi )^{2}}$.

2 The transverse impact parameter significance is defined as $d_0^\text {sig} = d_0 / \sigma _{d_0}$, where $\sigma _{d_0}$ is the uncertainty in the transverse impact parameter $d_0$.
